# The *MyoGravity* project to study real microgravity effects on human muscle precursor cells and tissue

**DOI:** 10.1038/s41526-024-00432-1

**Published:** 2024-10-03

**Authors:** Ester Sara Di Filippo, Sara Chiappalupi, Stefano Falone, Vincenza Dolo, Fernanda Amicarelli, Silvia Marchianò, Adriana Carino, Gabriele Mascetti, Giovanni Valentini, Sara Piccirillo, Michele Balsamo, Marco Vukich, Stefano Fiorucci, Guglielmo Sorci, Stefania Fulle

**Affiliations:** 1 Department of Neuroscience Imaging and Clinical Sciences, University “G. d’Annunzio” Chieti-Pescara, 66100 Chieti, Italy; 2 Interuniversity Institute of Myology (IIM), 06132 Perugia, Italy; 3 Department Medicine and Surgery, University of Perugia, 06132 Perugia, Italy; 4 Consorzio Interuniversitario Biotecnologie (CIB), 34127 Trieste, Italy; 5 Department of Life, Health and Environmental Sciences, University of L’Aquila, L’Aquila, Italy; 6 Agenzia Spaziale Italiana (ASI), Rome, Italy; 7 Kayser Italia S.r.l, Via di Popogna, 501, 57128 Livorno, Italy; 8 European Space Agency, Keplerlaan 1, NL-2200 AG Noordwijk, The Netherlands

**Keywords:** Physiology, Cell biology

## Abstract

Microgravity (µG) experienced during space flights promotes adaptation in several astronauts’ organs and tissues, with skeletal muscles being the most affected. In response to reduced gravitational loading, muscles (especially, lower limb and antigravity muscles) undergo progressive mass loss and alteration in metabolism, myofiber size, and composition. Skeletal muscle precursor cells (MPCs), also known as satellite cells, are responsible for the growth and maintenance of muscle mass in adult life as well as for muscle regeneration following damage and may have a major role in µG-induced muscle wasting. Despite the great relevance for astronaut health, very few data are available about the effects of real µG on human muscles. Based on the *MyoGravity* project, this study aimed to analyze: (i) the cellular and transcriptional alterations induced by real µG in human MPCs (huMPCs) and (ii) the response of human skeletal muscle to normal gravitational loading after prolonged exposure to µG. We evaluated the transcriptomic changes induced by µG on board the International Space Station (ISS) in differentiating huMPCs isolated from *Vastus lateralis* muscle biopsies of a pre-flight astronaut and an age- and sex-matched volunteer, in comparison with the same cells cultured on the ground in standard gravity (1×*g*) conditions. We found that huMPCs differentiated under real µG conditions showed: (i) upregulation of genes related to cell adhesion, plasma membrane components, and ion transport; (ii) strong downregulation of genes related to the muscle contraction machinery and sarcomere organization; and (iii) downregulation of muscle-specific microRNAs (myomiRs). Moreover, we had the unique opportunity to analyze huMPCs and skeletal muscle tissue of the same astronaut before and 30 h after a long-duration space flight on board the ISS. Prolonged exposure to real µG strongly affected the biology and functionality of the astronaut’s satellite cells, which showed a dramatic reduction of responsiveness to activating stimuli and proliferation rate, morphological changes, and almost inability to fuse into myotubes. RNA-Seq analysis of post- vs. pre-flight muscle tissue showed that genes involved in muscle structure and remodeling are promptly activated after landing following a long-duration space mission. Conversely, genes involved in the myelination process or synapse and neuromuscular junction organization appeared downregulated. Although we have investigated only one astronaut, these results point to a prompt readaptation of the skeletal muscle mechanical components to the normal gravitational loading, but the inability to rapidly recover the physiological muscle myelination/innervation pattern after landing from a long-duration space flight. Together with the persistent functional deficit observed in the astronaut’s satellite cells after prolonged exposure to real µG, these results lead us to hypothesize that a condition of inefficient regeneration is likely to occur in the muscles of post-flight astronauts following damage.

## Introduction

Microgravity (µG) refers to the near weightlessness environment associated with space flights. It arises as a consequence of the free-fall motion of the vehicle as it orbits the Earth or travels through space on a ballistic trajectory^1^. The human body undergoes profound changes in response to µG, most of which represent beneficial adaptations to the space environment. However, long-time exposure to µG induces alteration of the homeostasis in several organs and tissues with a negative impact on their functionality. Together with the bone, skeletal muscle is a dynamic tissue that adapts its density and structure to the forces induced by gravitational loading. In the presence of diminished mechanical load, skeletal muscles undergo alteration in the myofiber size and composition, due to an unbalance between protein synthesis and degradation, translating into a loss of muscle mass and strength (muscle atrophy)^2–4^.

After a space expedition, astronauts reported symptoms and conditions related to muscle wasting, with the peculiarity that this process occurs in less time than the age-related muscle atrophy experienced on Earth^5^. Significantly decreased volumes of *quadriceps* and *triceps surae* muscles have been reported after even a few days of space flight^6^. Microgravity-induced muscle wasting is comparable to that observed under denervation or immobilization conditions, which leads to a similar deterioration of muscle quantity and quality^7,8^.

The muscle atrophy experienced by crew members on board the International Space Station (ISS) is not efficiently prevented and counteracted by the mandatory execution of resistance physical exercises several hours a day^9^. This represents a major problem in view of long-duration space missions (such as those required to reach Mars) since muscle atrophy reduces astronauts’ motor function, and even a greater problem for the crew members when they come back to Earth from a space mission and have to readapt to the normal gravitational loading.

Weightlessness may also affect the functionality of satellite cells, the MPCs residing in a quiescent state between the basal lamina and the sarcolemma responsible for the growth and maintenance of muscle mass in adult life, as well as for muscle regeneration following damage (adult myogenesis)^10^. Once activated by stressful stimuli, these MPCs proliferate extensively and fuse with each other to form multinucleated myotubes, which start expressing proteins of the contractile machinery (including myosin light and heavy chains, troponins, and tropomyosins), leading to mature slow- or fast-twitch myofibers expressing specific myosin isoforms^11^. The activity of MPCs is under the control of both intracellular and extracellular factors that are spatially and temporally regulated. In particular, the myogenesis regulatory factors (MRFs), Myf5, MyoD, myogenin, and MRF4 are master transcription factors upregulated in differentiating MPCs and orchestrating the myogenesis process^12^. Moreover, muscle-specific microRNAs (miRNAs), also called myomiRs, play essential roles in myogenesis by regulating myoblast proliferation and differentiation^13^. A decline in the satellite cell function has been correlated with the reduced regenerative potential of muscles and muscle atrophy in several chronic diseases, muscle disuse, and during aging^14,15^, pointing to a key role of this cell type in µG-induced muscle wasting.

The objective of preserving the crew member’s health and fitness has fostered investigation into the molecular and cellular mechanisms underlying µG-associated muscle weakness, to develop nutritional/pharmacological approaches to preserve muscle mass during long-duration space flights. A wide range of in vitro and in vivo experimental models have been investigated under real or simulated µG conditions^16^. However, on-ground experimental models mimicking µG resulted not completely reliable, giving back divergent results from those obtained under real µG conditions. For example, differently from cast immobilization and bed rest models, in which fast-twitch (locomotor) and slow-twitch (postural) fibers undergo atrophy to a comparable extent, in real µG conditions, slow-twitch fibers resulted in more susceptible than the fast-twitch ones^3^. This is because traveling in space implies almost the absence of both the support reaction force and the direct effect of weight, generating a unique condition that is difficult to reproduce on the ground.

**Fig. 1:**
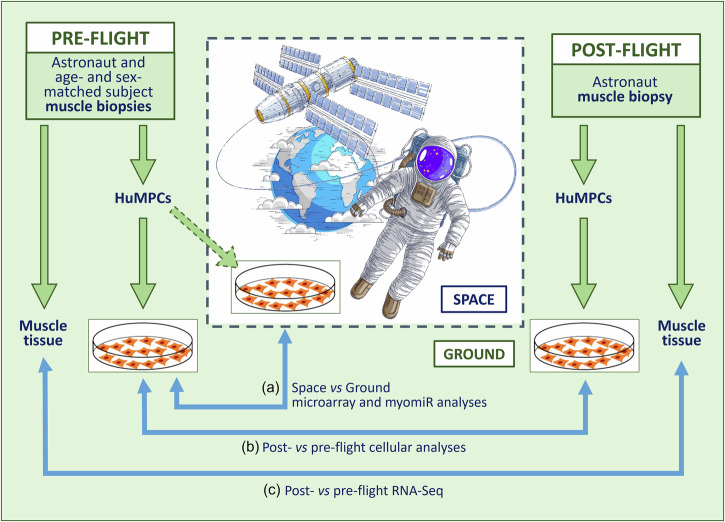
Scheme of MyoGravity experimental approach. (Licensed by Dreamstime; modified). HuMPCs isolated from muscle biopsies of an astronaut and an age- and sex-matched subject were cultured in standard gravity (1×*g*) or µG conditions on board the ISS, and comparative gene and myomiR expression analysis was performed (**a**). HuMPCs were isolated from Vastus lateralis muscle biopsies of the astronaut before and 30 h after a 5-month space mission and the age- and sex-matched subjects were analyzed for their biological properties (**b**). The astronaut’s pre- and post-flight muscle tissues underwent comparative transcriptome analysis (**c**).

The *MyoGravity* project, promoted and supported by the Italian Space Agency (ASI), aimed to evaluate the alterations induced by real µG on human muscle tissue and its MPCs to better understand the responses of skeletal muscles to unload, especially during long-duration space flights. To study the effects of real µG on muscle tissue and huMPCs, investigation at three different levels was performed involving a space mission crew member and an age- and sex-matched subject (Fig. 1): (i) microarray analysis of the gene expression profiles of huMPCs isolated from muscle biopsies of the astronaut before the space mission and the age- and sex-matched subject cultured in vitro on board the ISS or on the ground (Space vs. Ground); (ii) cellular analysis on huMPCs migrated out from muscle biopsies of the astronaut before (pre-flight) and 30 h after a long-duration (5 months) space mission (post-flight) and the age- and sex-matched subject; and (iii) RNA-Seq transcriptome analysis of the muscle biopsies obtained from the astronaut before (pre-flight) and after (post-flight) the space mission (Post- vs. Pre-flight) to assess the effects exerted in vivo by real µG on muscle tissue. Thanks to the peculiar timing at which the muscle biopsies were performed, we could have important information about the response of the astronaut’s muscle tissue to the reacquired Earth gravitational loading following a long-duration space flight. The main results of the *MyoGravity* project are reported in the present work.

## Results

### Prolonged exposure to real µG strongly affects the biology and functionality of huMPCs

**Fig. 2:**
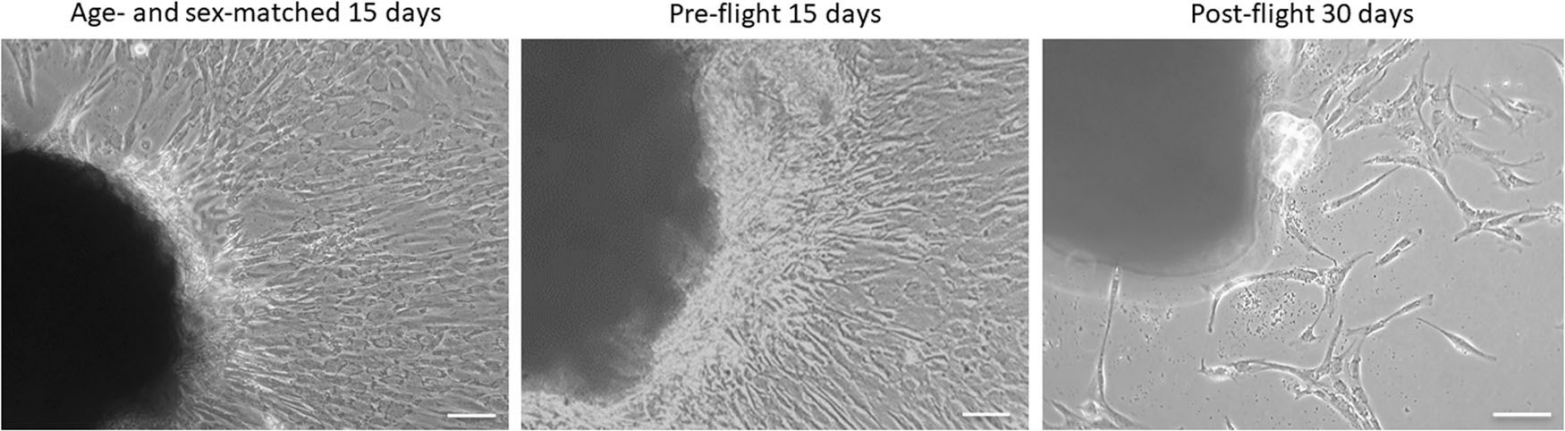
Representative images of huMPCs migrated out explants. Phase-contrast microscopy images of huMPCs migrated out from the VL muscle explants after 15 days for the age- and sex-matched subject and pre-flight astronaut and after 30 days for post-flight astronaut. Original magnification, ×20. Scale bar, 50 µm.

We obtained huMPCs after their migration from biopsies of *Vastus lateralis* (VL) muscles of pre- and post-flight astronauts and an age- and sex-matched volunteer who did not participate in any space mission. HuMPCs of the pre-flight astronaut and the volunteer migrated out of the explants on day 10 of culture, which is the time we usually observe with this kind of biopsies. Instead, huMPCs of the post-flight astronaut migrated out of the explants starting from day 28 and they were strongly reduced in number compared with the counterpart muscle biopsies (Fig. 2).

**Fig. 3:**
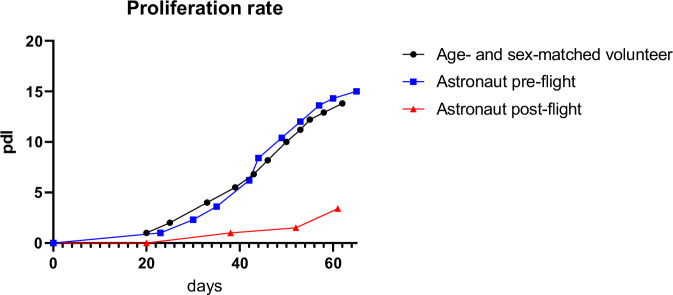
Proliferation rates of huMPCs. Duplication rate of huMPCs isolated from VL muscle biopsies of pre- and post-flight astronaut and the age- and sex-matched volunteer. Pdl, population doubling level.

The migrated huMPCs were detached, cultivated, and characterized for proliferation [population doubling level (pdl) number], myogenicity (percentage of desmin^+^ cells in growth medium, GM), and differentiation capability [fusion index (FI) and percentage of unfused myogenic cells at day 7 in differentiation medium, DM]. HuMPCs isolated from the post-flight muscle showed a strongly reduced pdl number over time compared with the pre-flight and volunteer ones (~3.5 vs. ~14.0 pdl at day 60 for post-flight and pre-flight/age- and sex-matched volunteer, respectively) (Fig. 3).

**Fig. 4:**
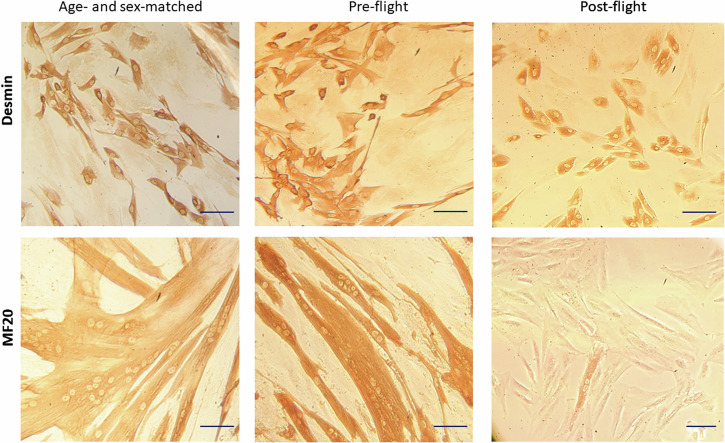
Characterization of huMPC cultures. Reported are representative images of huMPCs isolated from the age- and sex-matched volunteer and pre- and post-flight astronaut as viewed by phase-contrast microscopy after immunostaining for desmin in growth medium (GM) to evaluate myogenicity (*upper row*), or after immunostaining for MF20 at day 7 of culture in differentiation medium (DM) to evaluate the extent of myoblast fusion into multinucleated myotubes (*lower row*). Original magnification, ×20. Scale bar, 20 μm.

Moreover, while myoblast populations derived from the age- and sex-matched volunteer and pre-flight astronaut samples showed comparable myogenicity, post-flight huMPCs were characterized by a relatively low percentage of desmin^+^ cells, however sufficient to evaluate their differentiation potential (Fig. 4; Table 1). When induced to differentiate, volunteer and pre-flight huMPCs fused at a similar extent, with more than 50% nuclei found inside myotubes at day 7 in DM. On the contrary, post-flight huMPCs showed a substantial inability to fuse (only 1.2% myoblasts fused into myotubes), with rare and very small myotubes formed, and a concomitant huge increase in unfused desmin^+^ cells (Fig. 4; Table 1).

**Table 1:** Myogenicity (percentage of desmin^+^ cells) in GM, and fusion index and percentage of unfused desmin^+^ cells in DM were evaluated in huMPCs isolated from pre- and post-flight astronaut muscles and muscle of the age- and sex-matched volunteer

Samples	Desmin^+^ cells (%)	Fusion Index (%)	Unfused desmin^+^ cells (%)
Age- and sex-matched	56.4 ± 5.4	64.9 ± 9.1	26.0 ± 9.1
Pre-flight	62.4 ± 8.4	53.3 ± 17.6	40.9 ± 14.5
Post-flight	45.3 ± 6.2*	1.2 ± 0.1**	70.0 ± 1.0*

At least 500 cells were counted in 10 different randomly selected fields. Reported are the average values ± SD. Unpaired *t*-tests were used to reveal statistical differences.

*P < 0.05 and **P < 0.001, statistically significant post- vs. pre-flight.

**Fig. 5:**
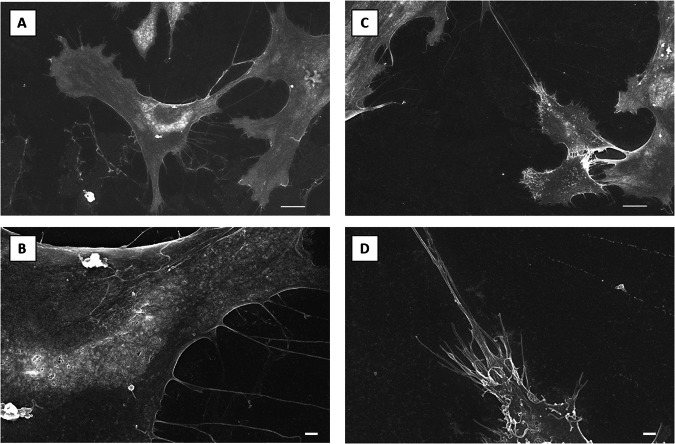
Electron microscopy images of astronaut’s pre- and post-flight huMPCs. Scanning electron microscopy representative images of huMPCs isolated from astronaut’s pre-flight (**A**, **B**) and post-flight (**C**, **D**) muscle biopsies. Scale bars, 10 µm (**A**, **C**); 1 µm (**B**, **D**).

Microgravity also affected huMPC morphology, as observed by scanning electron microscopy showing completely different patterns of membrane dynamics in huMPCs isolated from muscles before and after the space flight. Smooth membranes with relatively few and long filopodia were characteristics of pre-flight huMPCs, whereas rough membranes with multiple and short cytoplasmic elongations were observed in post-flight samples (Fig. 5).

### Microgravity downregulates genes involved in muscle structure and upregulates genes related to cell adhesion and ion transport

To have information about the effect of µG on the muscle differentiation process, huMPCs isolated from the pre-flight astronaut and age- and sex-matched subjects were cultivated inside the experiment units (EUs) on board the ISS for 7 days in DM. The temperature (*T*) profile from the integration of the EUs at the Kennedy Space Centre facilities to their insertion into the Kubik incubator on board the ISS was recorded (Fig. S1) in order to be reproduced during on-ground (1×*g*) control experiments, in which huMPCs of the same two subjects (at the same pdl numbers) and the EUs were used.

**Fig. 6:**
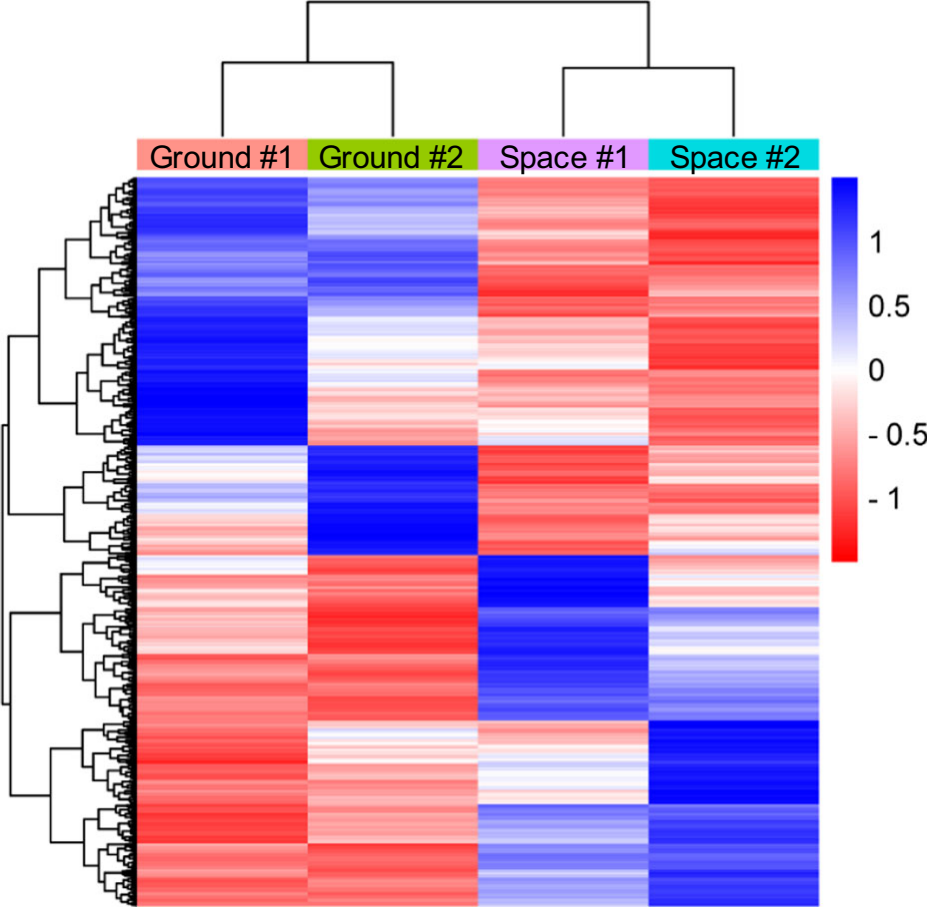
Hierarchically clustered heatmap of the differentially expressed genes. The Heatmap describes the gene expression trend in the two experimental conditions (Space vs. Ground; log2-fold change cut-off, 0.7) in huMPCs of pre-flight astronauts and the age- and sex-matched volunteers cultured on the ground (*Ground*) or in µG conditions on board the ISS (*Space*); Euclidean distance method. #1 and #2 indicate the subjects (astronaut or the volunteer) from whom huMPCs were derived.

Total RNAs of pre-flight astronaut's and volunteer's huMPCs cultured on board the ISS or on the ground were isolated at the end of the differentiation experiments and processed for microarray analysis to assess the gene expression profile. First, we considered genes that were downregulated or upregulated (Space vs. Ground) in common in the two subjects using 0.7 as a log2 fold change cut-off. We found a total of 1495 modulated, with 711 upregulated and 784 downregulated genes (Fig. 6).

Functional annotation analysis performed with the DAVID 2021 tool showed upregulated and downregulated genes as classified in 122 and 157 statistically significant chart records, respectively (Tables S1 and S2). Upregulated terms were mainly related to cell adhesion, plasma membrane components, and ion transport. Indeed, Pleckstrin homology-like domain, Synapse assembly, Phosphoprotein, Cadherin N-terminal, and Ion transport emerged as the most significant upregulated terms (Table S1). Seven protocadherins beta (*PCDHB-2*, *-5*, *-9*, *-10*, *-12*, *-14*, and *-16*) belonging to the cadherin superfamily, several voltage-gated channels (calcium, chloride, potassium, and sodium), and members of the solute carrier family appeared as the most represented upregulated genes (Table S3). Instead, Muscle protein, Structural constituent of muscle, and Sarcomere organization resulted as the most significant downregulated terms (Table S2). They included several myosin light and heavy chains, troponins, tropomyosin 3 (*TPM3*), and myomesins (Table 2). Cysteine and glycine-rich protein 3 (*CSRP3*), a positive regulator of myogenesis, and keratin 19 (*KRT19*), a protein that helps to organize costameres, appeared among the downregulated genes (Table 2). No terms related to muscle structure resulted from the analysis performed on the upregulated genes.

**Table 2:** Microarray analysis

Term: Muscle protein		
Gene ID	Gene name	Average fold change (SD)
72	actin gamma 2, smooth muscle(ACTG2)	−1.60 (0.72)
8736	myomesin 1(MYOM1)	−1.13 (0.07)
9172	myomesin 2(MYOM2)	−1.47 (0.70)
4629	myosin heavy chain 11(MYH11)	−2.58 (2.12)
4624	myosin heavy chain 6(MYH6)	−0.94 (0.13)
4625	myosin heavy chain 7(MYH7)	−1.43 (0.67)
4633	myosin light chain 2(MYL2)	−1.47 (0.41)
4634	myosin light chain 3(MYL3)	−3.48 (1.39)
4635	myosin light chain 4(MYL4)	−0.90 (0.24)
4636	myosin light chain 5(MYL5)	−0.92 (0.10)
7170	tropomyosin 3(TPM3)	−1.39 (0.38)
7125	troponin C2, fast skeletal type(TNNC2)	−1.36 (0.25)
7136	troponin I2, fast skeletal type(TNNI2)	−1.17 (0.30)

Term: Structural constituent of muscle		
Gene ID	Gene name	Average fold change (SD)
8048	cysteine and glycine-rich protein 3(CSRP3)	−2.41 (0.50)
3880	keratin 19(KRT19)	−1.80 (0.09)
8736	myomesin 1(MYOM1)	−1.13 (0.07)
9172	myomesin 2(MYOM2)	−1.47 (0.70)
4629	myosin heavy chain 11(MYH11)	−2.58 (2.12)
4633	myosin light chain 2(MYL2)	−1.47 (0.41)
4634	myosin light chain 3(MYL3)	−3.48 (1.39)
4636	myosin light chain 5(MYL5)	−0.92 (0.10)
8470	sorbin and SH3 domain containing 2(SORBS2)	−0.96 (0.06)
23336	synemin(SYNM)	−1.02 (0.19)
8557	titin-cap(TCAP)	−0.92 (0.23)

Term: Sarcomere organization		
Gene ID	GENE NAME	Average fold change (SD)
1482	NK2 homeobox 5(NKX2-5)	−0.91 (0.20)
8048	cysteine and glycine-rich protein 3 (CSRP3)	−2.41 (0.50)
3880	keratin 19(KRT19)	−1.80 (0.09)
442721	leiomodin 2(LMOD2)	−0.84 (0.04)
9172	myomesin 2(MYOM2)	−1.47 (0.70)
4624	myosin heavy chain 6(MYH6)	−0.94 (0.13)
4625	myosin heavy chain 7(MYH7)	−1.43 (0.67)
51778	myozenin 2(MYOZ2)	−1.21 (0.30)
8557	titin-cap(TCAP)	−0.92 (0.23)

Most significant terms of functional annotation chart among the genes downregulated in common (Space vs. Ground; log2fold change cut-off, 0.7) in huMPCs isolated from the pre-flight astronaut and age- and sex-matched volunteer.

Of note, none of the myogenic regulatory factors (MRFs), MyoD (*MYOD1*), Myf5 (*MYF5*), myogenin (*MYOG*), and MRF4 (*MRF4*) appeared in the list of the genes modulated (0.7 log2-fold change cut-off) in common. Indeed, these muscle-specific transcription factors were differently modulated in the two subjects, with only *MYOD1* slightly upregulated in huMPCs isolated from both the pre-flight astronaut and the volunteer (1.21 and 0.08 log2 fold change, respectively) (data not shown).

Interestingly, WebGestalt gene set enrichment analysis (GSEA) performed on genes modulated in common (0.7 log2 fold change cut-off) showed two categories related to catabolic processes as significantly upregulated in huMPCs differentiating under µG conditions (Fig. S2; Table S4), suggesting that unloading also induces atrophy in newly formed myotubes.

To unravel the genes mainly affected by µG, we performed functional annotation analysis using 1.5 as a log2 fold change cut-off. In this case, we found only 83 genes modulated in common, of which 21 upregulated and 62 downregulated genes (Tables S5, S6). The list of modulated genes included 11 unknown genes. Channel activity, Cell membrane, Insulin-like growth factor binding protein, Cell projection, and Postsynaptic membrane emerged as the most significant upregulated terms (Table S5). Genes encoding for the Eph receptor B2 (*EPHB2*), and the member of the ferlin family, otoferlin (*OTOF*), both of which are implicated in muscle differentiation and myoblast fusion^17,18^, resulted as the most represented upregulated genes. Among the most significant downregulated terms were Receptor binding, Inflammatory response, Transcriptional repressor activity, and several terms made up genes related to muscle structure and contraction, especially *CSRP3* (average log2 fold change, −2.41), *KRT19* (average log2 fold change, −1.80), and myosin light chain 3 (*MYL3*; average log2 fold change, −3.48) (Table S6).

### Microgravity downregulates the expression of muscle-specific microRNAs

**Fig. 7:**
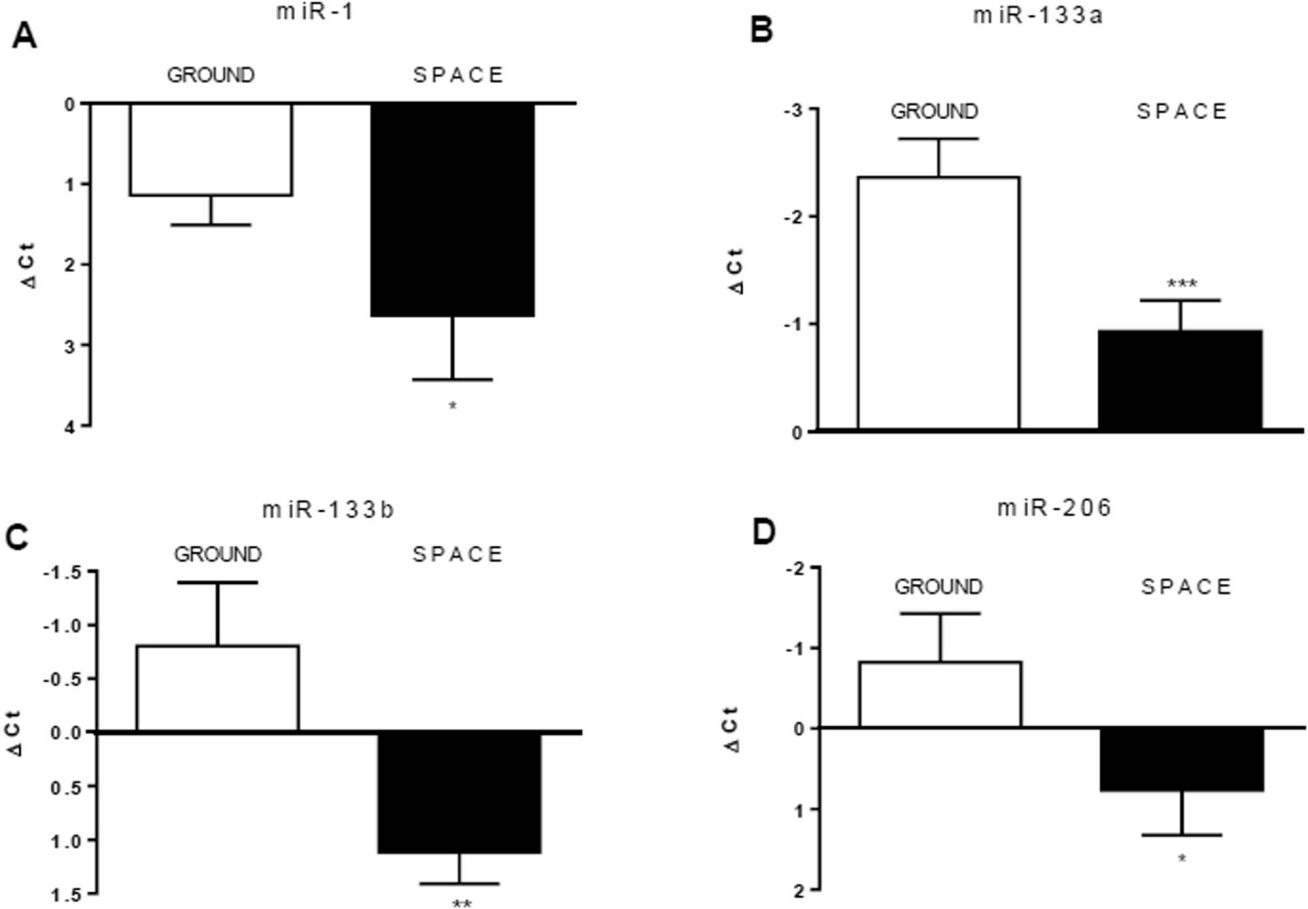
The expression levels of the myomiRs. miR-1 (**A**), miR-133a (**B**), miR-133b (**C**), and miR-206 (**D**) were evaluated in huMPCs isolated from the pre-flight astronaut and age- and sex-matched volunteer cultured in 1×*g* (*Ground*) or µG (*Space*) conditions. The ubiquitous miR-16 was used as a housekeeping gene to normalize miRNA levels. Results are shown as ΔCt [Ct_(miRNA-of-interest)_−Ct_(miR-16)_] and are the means ± SD of three independent experiments, each performed in triplicate. Unpaired *t*-test was used to reveal statistical differences. *P < 0.05, **P < 0.01 and ***P < 0.0001, statistically significant.

We analyzed differentiating huMPCs cultured on the ground or on board the ISS for the expression of the main muscle-specific myomiRs sustaining myoblast proliferation or differentiation and involved in muscle atrophy (Table S7)^13,19^. We found that miR-1, miR-133a, miR-133b, and miR-206, were downregulated in µG conditions in both the volunteer- and pre-flight astronaut-derived cells (Fig. 7). This suggested an overall impairment of huMPC functionality induced by unloading and potentially predisposing to muscle atrophy, in accordance with the results obtained by the gene expression analysis (Fig. S2).

### Genes involved in muscle remodeling are promptly activated after landing following a long-duration space flight

**Fig. 8:**
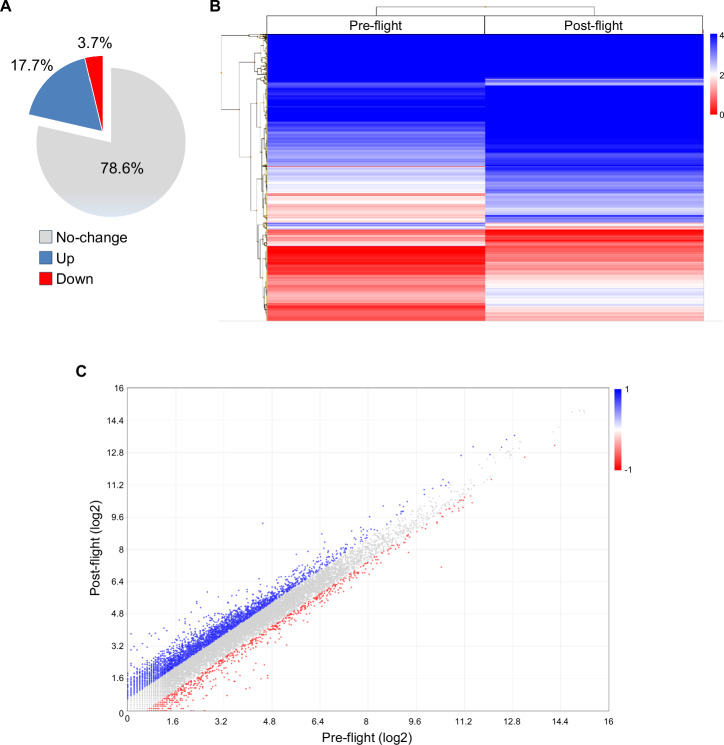
RNA-Seq analysis. It performed in duplicate in post- vs. pre-flight astronaut’s muscle sample using the Transcriptome Analysis Console Software, version 4.0.1 (fold change cut-off, 1.5). **A** The percentages of unchanged, upregulated, or downregulated genes are reported. **B** Hierarchically clustered heatmap of differentially expressed genes (log2 values) in post- vs. pre-flight muscle sample. The transition from red to blue strips represents an increase in gene expression levels. **C** Scatter plot of transcripts differentially expressed (log2 values) in the two experimental groups. Red and blue dots represent significantly downregulated and upregulated genes, respectively. ANOVA method (eBayes-corrected variance) was used to reveal statistical differences. *P*-value < 0.05 (**A**–**C**).

Besides the isolated huMPCs, we had the opportunity to analyze the transcriptome of an astronaut’s muscle tissue before (pre-flight) and 30 h after (post-flight) a 5-month space mission by performing RNA-Seq analysis of the relative VL muscle biopsies. We analyzed a total of 20,824 genes and found 4454 modulated (post- vs. pre-flight; fold change cut-off, 1.5; *P*-value < 0.05) genes, of which 3679 (82.6%) upregulated and 775 (17.4%) downregulated (Fig. 8).

**Fig. 9:**
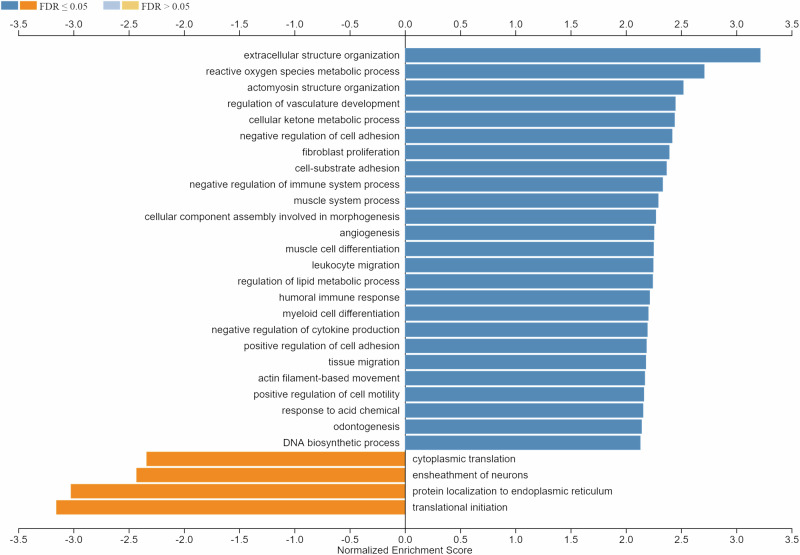
WebGestalt gene set enrichment analysis. GSEA (functional category, Biological Process-noRedundant) in post- vs. pre-flight astronaut’s muscle sample (fold change cut-off, 1.5). Minimum number of genes in the category, 20. Shown are the resulting significant categories (FDR < 0.05).

To get information about the main biological processes affected by µG in muscles, we performed GSEA with WebGestalt. No. 4125 genes mapping to unique entrez gene ID were recognized as unambiguous by the tool, and 3213 genes were annotated to the selected functional categories. Interestingly, genes related to extracellular matrix, cell adhesion, and reactive oxygen species metabolic process emerged among the upregulated terms together with several terms related to muscle structure, contraction, and differentiation (Fig. 9). Downregulated terms included genes related to the translation process, endoplasmic reticulum, and neuron myelin ensheathment (Fig. 9).

**Fig. 10:**
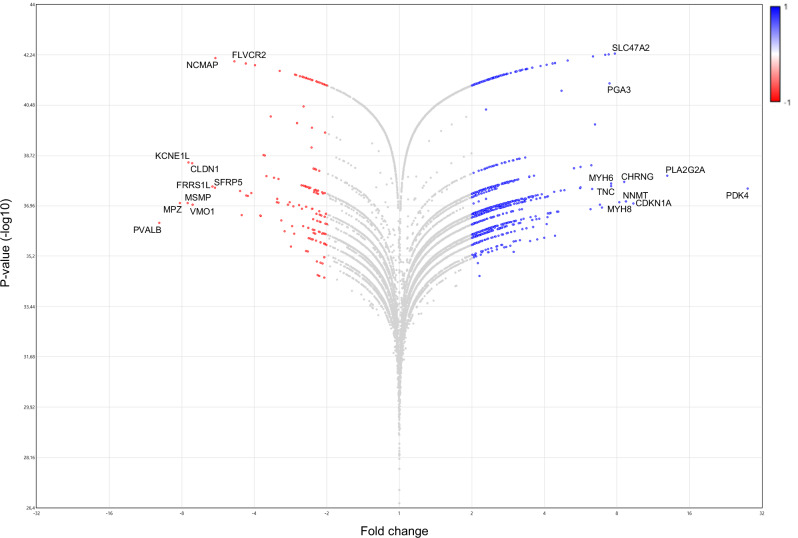
Volcano plot. This figure shows differentially expressed genes (Fold change vs. *P*-value [-log10]) in post- vs. pre-flight astronaut muscle samples obtained by the Transcriptome Analysis Console Software, version 4.0.1 (fold change cut-off, 2.0). ANOVA method (eBayes-corrected variance) was used to reveal statistical differences. *P*-value < 0.05. Red and blue dots represent significantly downregulated and upregulated genes, respectively. The ten most downregulated or upregulated genes are indicated.

To have information about the most altered genes and biological processes, we performed analysis setting 2.0 as a fold change cut-off (post- vs. pre-flight) and obtained 1223 modulated genes, of which 1075 (87.9%) upregulated and 148 (12.1%) downregulated. By DAVID GO analysis we found 224 statistically significant chart records relative to the upregulated genes (Table S8), and 15 statistically significant chart records relative to the downregulated genes (Table S9). It is to be noted that Extracellular matrix, Cell adhesion, and Integrin binding continue to emerge as the most significant records for upregulated genes (Table S8). They include transforming growth factor (TGF)-β1 and -β2, several isoforms of collagen (i.e., *COL1A1*, *COL3A1*, *COL4A-1*, *-2* and *-5*, *COL5A2*, *COL6A-1* and *-2*, *COL16A1*, *COL18A1*, *COL19A1*), and several integrin alpha subunits (*ITGA-2*, *-3*, *-5*, *-8*, *-9*, and *-10*, and *ITGAM*) (Table S10). Upregulated genes also include members of the cadherin family [i.e., *CDH4, CDH15*, FAT atypical cadherin 1 (*FAT1*), *PCDHB7*, and *PCDHB13*], NCAM (*NCAM1*), and *IGF1* (Table S10). Several classes referred to myosins and other muscle proteins also emerged among the statistically significant upregulated chart records (Table S8), including genes of myosin light and heavy chains, actins, troponins, calsequestrin 2 (*CASQ2*), and tropomyosin 4 (*TPM4*) (Table S10). *MYH8* resulted as the most upregulated myosin gene, and the sixth most upregulated gene in absolute (fold change, 8.17; *P*-value, 4.67E−36) (Fig. 10). Although no terms related to oxidative stress emerged by this analysis, pyruvate dehydrogenase kinase 4 (*PDK4*), a mitochondrial enzyme involved in the oxidative decarboxylation of pyruvate, resulted as the most upregulated gene in post- vs. pre-flight muscle (fold change, 27.89; *P*-value, 2.7E−38) (Fig. 10).

Downregulated chart records included genes involved in Cell-cell adhesion via plasma-membrane adhesion molecules, Calcium ion binding, epidermal growth factor (EGF), extracellular space, and positive regulation of bone mineralization (Table S9). The cell adhesion molecules, cadherins 7 (*CDH7*), 19 (*CDH19*), and 20 (*CDH20*), and protocadherin 8 (*PCDH8*) appeared in the most significant downregulated term (Table S11). The gene encoding for myelin protein zero (*MPZ*; fold change, −8.16; *P*-value, 8.62E−38) resulted as the second most downregulated gene (Fig. 10; Table S11). The S100B protein (*S100B*; fold change, −3.97; *P*-value, 1.31E−42), neurexin 1 (*NRXN1*; fold change, −2.34; *P*-value, 2.56E−38), teneurin transmembrane protein 2 (*TENM2*; fold change, −3.14; *P*-value, 2.05E−42), neuregulin 4 (*NRG4*; fold change, −2.2; *P*-value, 4.94E−42), and parvalbumin (*PVALB*; fold change, -9.94; *P*-value, 4.36E−37) also appeared in the most significant downregulated terms (Table S11). In particular, *PVALB*, which has been reported to promote muscle wasting, resulted as the most downregulated gene in post- vs. pre-flight muscle samples (Fig. 10). However, bone morphogenetic protein 7 (*BMP7*; fold change, −3.17; *P*-value, 1.25E−38) and matrilin 2 (*MATN2*; fold change, −2.44; *P*-value, 1.18E−37) (Table S11), both of which sustain muscle hypertrophy^20,21^, resulted also downregulated in post-flight muscle tissue.

## Discussion

Long-duration space-flight missions represent a challenge for the international aerospace community since, under µG conditions, crew members’ bodies undergo relevant adaptations and perturbation of their homeostasis, often resulting in loss of functionality. Skeletal muscle is one of the most affected tissues, due to its plasticity to adapt its myofiber size, metabolic properties, and vascularization to changing environments^22^. For this reason, skeletal muscle has been the object of intense investigation in different gravitational conditions, on board the ISS or simulated on the ground, in which muscle cells or different animal species (mostly, mice) have been studied to elucidate the mechanisms underlying µG-induced muscle wasting^23^. However, very few data relative to huMPCs and human muscle tissue are available.

We had the opportunity to evaluate the transcriptomic changes occurring in the skeletal muscle tissue of a crew member following a long-duration space flight on board the ISS. At the same time, we were able to evaluate the transcriptomic changes induced by real µG in differentiating huMPCs previously isolated from muscle biopsies of the same crew member and an age- and sex-matched volunteer (Fig. 1). First, we found that persistent µG conditions strongly affect the biological properties and the myogenic potential of in situ huMPCs since long-term space flight translated into a dramatic reduction of the satellite cell responsiveness to activating stimuli (as evidenced by the longer migration time out of the post-flight biopsy; Fig. 2), and reduced proliferation rate and almost inability of activated myoblasts to fuse into myotubes once exposed again to standard gravity conditions (Figs. 3, 4; Table 1). A similar deficit in the fusion process was reported with rat L8 myoblasts after 9 days of culture on board a space shuttle^24^. The reduced number of huMPCs migrated out of the post-flight biopsy also suggests that a loss of myofiber-associated satellite cells can occur during long-term space flights. The morphological results suggest that exposure to µG has a deep impact on the biology of huMPCs, which can likely reflect on membrane alterations in terms of cell–cell interactions, cell–matrix anchorage, and the release of extracellular vesicles. This aspect deserves more investigation.

The microarray analysis performed on huMPCs cultured on board the ISS in comparison with the same cells cultured on the ground gave us important information about the acute effects of real µG on the myogenic differentiation process. Upregulated genes belong to terms related to cell adhesion, plasma membrane components, and ion transport. Pleckstrin homology-like domain, which appeared as the most significant upregulated term, is implicated in protein interactions at the interface between the plasma membrane and the cytoskeleton^25^. Seven protocadherins beta, which are involved in cell–cell interactions^26^, resulted in upregulation by µG in differentiating huMPCs of both the investigated subjects, suggesting a major role of these proteins in modulating myoblast differentiation. The observed upregulation of genes involved in cell–cell interaction or ion transport may represent a compensative response of huMPCs undergoing differentiation in µG conditions.

Noteworthy, a strong downregulation of genes related to the contraction machinery and sarcomere organization was observed in huMPCs differentiating on board the ISS. These genes include several myosin light and heavy chains and troponins. Also downregulated were the myomesins *MYOM1* and *MYOM2*, and *KRT19*, which play a role in the assembly of sarcomere and costamere organization, respectively^27,28^. These results highlight defects in the maturation phase of myotube formation in the absence of gravitational loading.

Cysteine and glycine-rich protein 3 (*CSRP3*) (also known as muscle LIM protein; MLP) resulted as one of the most downregulated genes in huMPCs differentiating in µG conditions (Table 2). Interestingly, *CSRP3* is a promoter of myogenic differentiation through direct interaction with the MRFs^29^, and *CSRP3* silencing was reported to impair C2C12 myoblast differentiation, with reduced expression of myogenin and MyoD mRNAs, leading to disorganized and reduced in number myotubes^30^. However, we did not find concordant deregulation of myogenin (*MYOG1*), and MyoD (*MYOD1*) was slightly upregulated in differentiating huMPCs of the astronaut and the volunteer. Also downregulated appeared *EPHB2*, which is implicated in muscle differentiation and has a potential role in satellite cell-mediated muscle repair^18^, and *OTOF* (otoferlin), which has a well-characterized role in myoblast fusion^17^.

Altogether, these data suggest that unloading characterizing µG conditions mainly affects the building of the contractile apparatus in differentiating myoblasts and newly-formed myotubes rather than the basal transcriptional activity typical of the myogenic differentiation process.

The muscle-specific miRNAs, miR-1, miR-133a, miR-133b, and miR-206, which enhance myoblast differentiation and are dysregulated in muscle atrophy conditions and aged huMPCs^19,31^, resulted downregulated in huMPCs differentiating on board the ISS (Fig. 7). This is in line with the observed downregulation of genes related to the contraction machinery (Table 2) and the concomitant activation of catabolic pathways (Fig. S2; Table S4). miR-1 has been reported to also promote muscle differentiation by restraining histone deacetylase 4 (HDAC4) expression and strengthening MEF2 activity^32^. In accordance, we found upregulated *HDAC4* in huMPCs differentiated on board the ISS (0.78 ± 0.43 average log2 fold change Space vs. Ground) (data not shown). Additional investigation is necessary to assess the specific role of the investigated myomiRs in the skeletal muscle wasting associated with exposure to real µG.

Since the changes observed on isolated huMPCs do not necessarily reflect what happens in vivo under µG conditions, we performed additional investigation on muscle biopsies isolated from the astronaut before and 30 h after landing from his/her long-term space mission. RNA-Seq analysis of post- vs. pre-flight muscle tissue showed several upregulated genes as mainly related to the extracellular matrix and cell adhesion, including TGF-β1 and -β2, several collagen types, and several integrin alpha subunits (Table S10). A decreased expression of collagens has been reported in the VL muscles of subjects experiencing severe muscle atrophy obtained with a dry immersion model characterized by drastic hypoactivity^33^. Since collagens play a critical role in muscle structural integrity and force transmission, their increased expression in post-flight muscle may represent a response finalized to rescue the muscle function lost during the space mission.

Many upregulated (post- vs. pre-flight) genes are involved in skeletal muscle differentiation and regeneration. Among them, integrin alpha 3 subunit (*ITGA3*), integrin alpha 5 subunit (*ITGA5*), and cadherin 15 (CDH15; also known as muscle cadherin, M-cadherin) are expressed in myoblasts or myotubes during the differentiation process and/or muscle development^34–36^; and, integrin alpha M subunit (*ITGAM*; also known as CD11B or Mac-1) is increased in macrophages of skeletal muscles in older adults treated with neuromuscular electrical stimulation combined with protein supplementation during bed rest^37^. Moreover, IGF1, a growth factor known to promote muscle hypertrophy and whose expression is increased in murine muscles in real µG conditions^38^, was upregulated in post- vs. pre-flight muscle. Similarly upregulated was NCAM (*NCAM1*), a multifunctional cell-surface protein expressed in quiescent satellite cells (for which it represents a marker) and proliferating/differentiating myoblasts and associated with muscle regeneration^39,40^. NCAM is also involved in the development and plasticity of neuromuscular junctions (NMJ)^41^, suggesting a potential µG-induced rearrangement of muscle innervation, to some extent.

*MYH8*, which corresponds to a developmental myosin expressed in neonatal muscles and during muscle regeneration^42^ and has been found highly upregulated after bed rest^43^, emerged as one of the most upregulated genes 30 h after landing. Together with the upregulation of other muscle contractile components, including myosin light and heavy chains, actins, troponins, and *CASQ2* and *TPM4* (Table S10), the increased expression of *MYH8* suggests a scenario reminiscent of muscle regeneration. Since *MYH8* is more abundant in fast-twitch fibers^44^, its increased expression in post-flight compared with pre-flight muscle may also be related to the shift from slow- to fast-twitch fiber types induced by µG during even short space flights^45^. *MYH6* and *MYH7*, which are typical of type I (slow-twitch) myofibers and whose absence determines a fast-twitch phenotype^44^, resulted also upregulated in post-flight muscle. Since post-flight biopsy was obtained from the astronaut 30 h after landing, these results suggest that genes involved in muscle remodeling are promptly activated following a long-duration space flight and that the shift from slow- to fast-twitch fiber types induced by exposure to µG is rapidly counterbalanced after landing by re-expression of *MYH6* and *MYH7*.

The most downregulated gene in post- vs. pre-flight conditions is parvalbumin (*PVALB*) (Fig. 10), a calcium-binding protein expressed in fast-twitch myofibers and whose expression increases upon denervation or unloading^46,47^. Thus, downregulating *PVALB* expression appears as a muscle response in favor of hypertrophy when µG-adapted skeletal muscles are re-exposed to the normal gravitational loading.

However, the changes observed in the expression of several genes suggest a condition of altered muscle homeostasis and compromised muscle innervation following a space mission. Indeed, (i) *PDK4*, which has been reported to impair mitochondrial function mediating the effects of prolonged muscle inactivity (e.g., muscle protein degradation and atrophic gene upregulation)^48^, resulted as the most upregulated gene (Fig. 10); (ii) myelin protein zero (*MPZ*) and the calcium-binding protein S100B (*S100B*), which are involved in peripheral nerve myelination^49,50^, were among the most significant downregulated terms, with MPZ resulting as the second most downregulated gene; and, (iii) neurexin 1 (*NRXN1*), teneurin transmembrane protein 2 (*TENM2*), and neuregulin 4 (*NRG4*), which have been implicated in synapse and neuromuscular junction (NMJ) organization^51–53^, resulted also downregulated in post-flight muscle tissue. Since S100B is also expressed in myoblasts and has a role in stimulating or inhibiting myogenesis and muscle regeneration depending on the myoblast density and S100B concentration^54^, we cannot exclude that the reduced expression of this protein may also be related to the huMPC component. This aspect deserves further investigation.

A previous study evaluating the serum biochemical profiles of an astronaut during his 1-year space mission and a set of 28 astronauts returning from 6-month missions showed an increase in cytokines associated with muscle regeneration immediately after landing on Earth^55^. Based on the cytokines found increased, which included CC chemokine ligand 2 (CCL2), interleukin (IL)-6, IL-10, IL-1 receptor antagonist, and C-reactive protein, the authors concluded that a signature of muscle regeneration after atrophy rather than a detrimental inflammatory response occurs after landing from a long-duration space flight^55^. Our data from the analysis of pre- and post-flight muscle tissues strongly support this conclusion. In line, *CCL2*, which has been reported to positively regulate satellite cell activation and proliferation^56^ and to promote muscle repair by inducing the upregulation of IGF1 expression^57^, was found upregulated (fold change, 2.16; *P*-value, 1.11E−36) (not shown) together with *IGF1* itself (Table S10) in post- vs. pre-flight muscle.

Altogether, our results reveal a compromised differentiation process in isolated huMPCs under real µG conditions, characterized by the downregulation of genes involved in muscle structure and sarcomere organization together with genes implicated in muscle differentiation, fusion into myotubes, and muscle repair. Moreover, a persistent functional deficit and a possible reduction in numbers are induced in the astronaut’s muscle satellite cells by prolonged exposure to µG on board the ISS, leading us to speculate that a condition of inefficient regeneration is likely to occur following damage during a space flight and even after landing. Finally, our data indicate that skeletal muscles rapidly readapt their contractile machinery to the normal gravitational loading after returning to Earth following a long-duration space flight, although they appear not able to promptly recover the physiological myelination/innervation pattern. This latter aspect should be taken into particular consideration when defining approaches to counteract muscle wasting in astronauts involved in long-duration space missions, and rehabilitation protocols after landing.

Although further research on additional crew members should be performed to confirm our data, the information obtained from this pilot study about the changes in human MPCs and muscle tissue in response to µG could drive the design of nutritional or pharmacological interventions to counteract the muscle alterations experienced by astronauts during long-term space missions. This knowledge could also be useful in establishing clinical approaches to muscle atrophy conditions occurring on Earth, such as denervation, aging, and disuse, since they share molecular mechanisms and signaling pathways with µG-induced muscle wasting.

### Strengths and limitations

This study evaluated the alterations induced by real µG on muscle tissue and huMPCs of the same subject. This allowed us to make correlations between the alterations observed in human skeletal muscle tissue and its own MPCs through the analysis of pre- and post-flight muscle biopsies, huMPCs isolated from pre- and post-flight muscles, and isolated huMPCs differentiating on board the ISS.

The main limitation of the study is represented by the availability of only one to investigate the alterations of muscle transcriptomic profile and huMPC morphology and biology in dependence on real µG (post- vs. pre-flight). Similarly, the limited number of slots in the Kubik incubator made available to us on board the ISS allowed us to cultivate huMPCs of two subjects at maximum (i.e., the astronaut and age- and sex-matched subject) to evaluate the effects of real µG on myoblast differentiation process (Space vs. Ground). In this latter case, we focused on the alterations in gene expression that occurred in common in the two subjects to bypass the interindividual variability.

Another limitation of the study is represented by the timing of the post-flight muscle biopsy collection, which was performed 30 h after landing, when the astronaut, even though he/she had not resumed normal motor activities, had experienced again the effects of Earth’s gravity for several hours. However, the particular timing of this muscle biopsy allowed us to observe the prompt response of the muscular system to the reacquired gravitational loading after landing from a long-duration space flight.

Moreover, it has to take into account that uncontrolled confounders, such as the diet composition and supplementation, possible pharmacological treatments, the physical exercise protocol followed, and the degree of radiation exposure could have modulated the real effects of µG on the astronaut’s muscles. The in vitro experiments carried out on the ISS, on the other hand, may have been only minimally affected by radiation exposure since huMPCs were contained within EUs integrated inside the KIC-SL and positioned within the ESA Kubik facility, representing multiple shields against radiations.

## Methods

### Samples

Muscle biopsies of *Vastus lateralis* (VL) muscle of the same astronaut were performed 2 months before his long-term (~5 months) space mission on board the ISS (pre-flight) and 30 h after his return to Earth (post-flight). Biopsy of VL muscle of an age- and sex-matched subject was also performed. The subjects gave informed consent for inclusion before participating in the study. The protocol for the astronaut biopsies was approved by ESA MB (prot. no. 2016_06_05). The protocol of the MyoGravity project was also approved by NASA (prot. no. Pro2013, NASA MPA no. NASA 7116301606HR). Pre- and post-flight biopsies were performed using the rongeur technique^58^ upon approval by the European Space Agency (ESA) MB (prot. no. 2016_07_01). A rongeur forceps were used for skeletal muscle tissue sampling through a ~1 cm skin/fascia incision following local anesthesia (2 ml of 1% lidocaine). Samples were subdivided into several tissue pieces (~20 mg each) for cellular and molecular analyses. The age- and sex-matched subject was enrolled at the University of Chieti-Pescara (Chieti, Italy) and underwent percutaneous needle biopsy^59^. This study was approved by the Ethics Committee for Biomedical Research, University of Chieti (No. 04 of 25.02.2016, and No. 22 of 01.12.2016), and complied with the Declaration of Helsinki. The inclusion criteria were normal ECG and blood pressure and absence of metabolic, cardiovascular, chronic bone/joint, or muscular diseases.

### Cell cultures

To obtain huMPCs, all VL muscle biopsies were processed according to the previously reported procedures^59^^,60^. Briefly, the tissue samples were collected in a sterile manner using tweezers and were placed into 10 ml amounts of sterile medium containing HAM’s F-10 medium (Euroclone, Milan, Italy) supplemented with 50 mg/ml gentamycin, at 4 °C for a maximum of 4 h and then, frozen in liquid nitrogen in 1 ml fetal bovine serum (FBS) plus 10% dimethyl sulfoxide (Sigma-Aldrich, Milan, Italy), and stored until further use. In order to obtain the explants, the samples were rapidly defrosted at 37 °C, and tissue fragments were manually minced and cultured in plates. Ten to thirteen days later, the first mononucleated cells migrated out of explants and were removed. For cell growth, huMPCs were cultured in HAM’s F-10 (Euroclone) supplemented with 0.1% gentamycin and 20% Defined Hyclone FBS (Euroclone) (growth medium; GM). At the first cell passage when explants were removed, cell populations were taken as population doubling level (pdl) = 1. The pdl number was calculated with the equation log_10_ (*N*/*n*)/ln_2_, with *N* as the number of cells at the time of detachment and *n* as the number of cells initially seeded. In order to induce differentiation into myotubes, huMPCs cultured in GM were switched to a differentiation medium (DM)^61^.

### Immunocytochemistry assays

Myogenicity and differentiation capability of huMPC cultures were analyzed using immunocytochemistry assays^61^. To determine the myogenic purity, the cells were labeled with an anti-desmin primary antibody (D33 monoclonal mouse anti-human desmin) (Dako, Glostrup, Denmark), using the Dako Real Peroxidase/DAB+ detection system (Dako). Ten random photos were taken for each well with a digital camera (Canon EOS 350D, Tokyo, Japan) connected to a light microscope at magnification ×20. The ImageJ software (ImageJ v1.8.0_172, National Institutes of Health, USA) was used to count at least 500 cells per sample. Myogenicity was expressed as the percentage of desmin^+^ cells. Counts of desmin^+^ cells after 7 days in DM were performed to evaluate the percentages of unfused myogenic cells.

The fusion index was calculated after labeling the cells with an anti-myosin heavy chain monoclonal antibody (MF20; Developmental Studies Hybridoma Bank, Iowa City, IA). The fusion index was expressed as the percentage of nuclei inside myotubes versus the total nuclei, with myotubes defined as cells positive for myosin-heavy chains containing 2 or more nuclei. Ten randomly chosen fields at magnification ×20 were counted for each sample.

### HuMPC culture in the Experiment Units

**Fig. 11:**
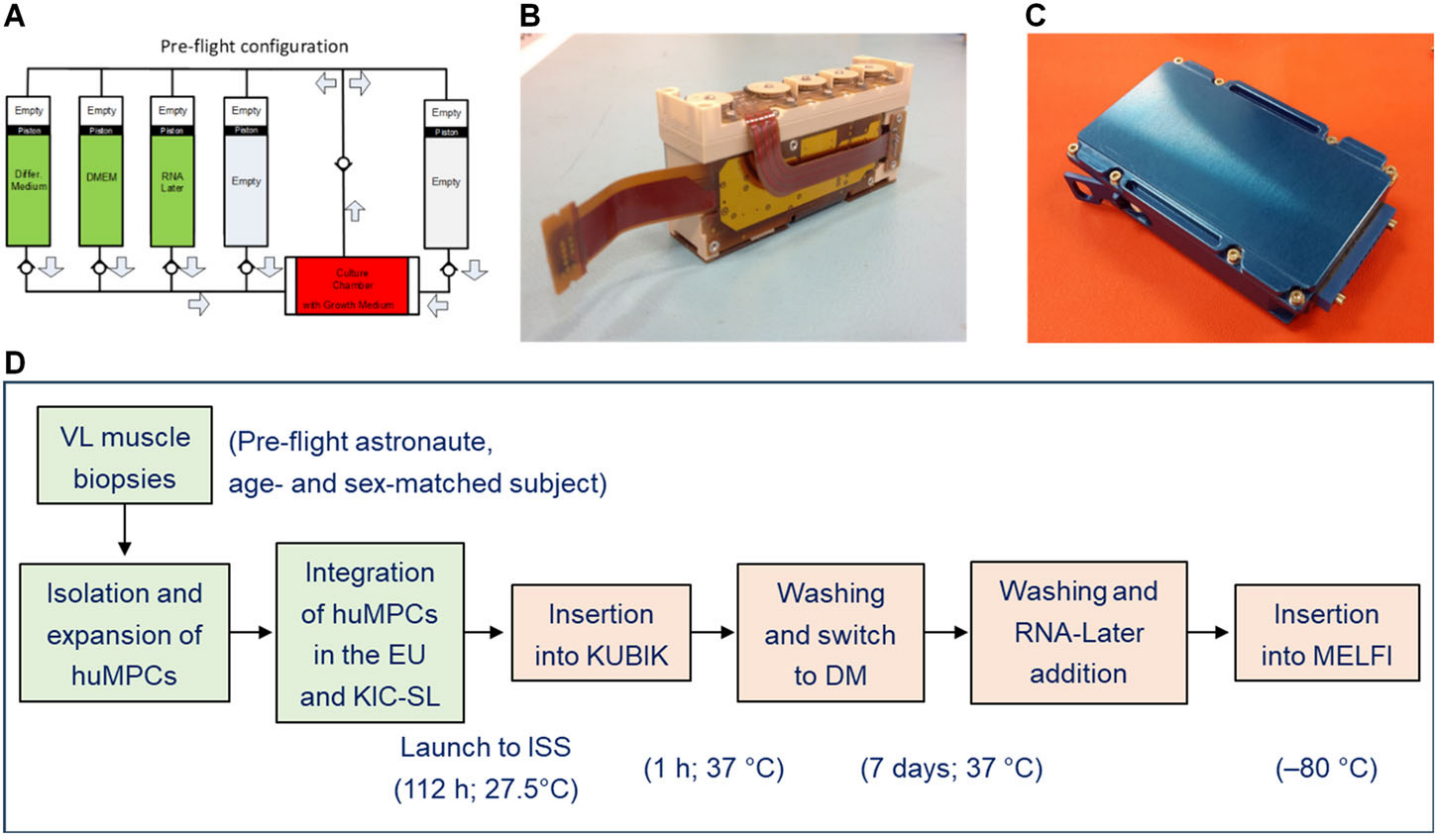
EU device and schematic steps of the experiment on board the ISS. **A** Schematic of the fluidic circuit of the KEU-ST Experiment Unit (EU) as loaded for the flight. **B** The EU is fully assembled with control electronics. **C** The KIC–SL interface container is fully assembled. **D** Schematic of the steps characterizing the experiments with huMPCs differentiating on board the ISS. Reported are the lengths and temperatures registered for each step. HuMPCs of the same subjects at the same pdl numbers were cultured in the EUs at the same times and temperature conditions in parallel experiments performed on the ground. VL vastus lateralis.

HuMPCs of pre-flight astronauts and age- and sex-matched healthy subjects were cultured in DM for 7 days to induce myotube formation on board the ISS (µG) or on the ground (1×*g*) for comparison. To have the most reliable results, cells with the same pdl numbers were used in both sets of experiments (astronaut, 8.00 ± 0.37 pdl; age- and sex-matched subject, 8.22 ± 0.30 pdl). For both on-ground and ISS experiments, huMPCs were seeded on Thermanox™ coverslips (4.0 × 10^5^ cells) inside dedicated experiment units (EUs) developed by Kayser Italia (KEU-ST; Fig. 11), as previously described^61^.

EUs were composed of a cell culture chamber and five cylindrical reservoirs (Fig. 11A), which were filled with the following media and chemicals: GM + HEPES 20 mM (culture chamber); DM + HEPES 20 mM (Reservoir 1); DMEM + HEPES 20 mM (Reservoir 2); RNA-Later Solution (AM7020 Invitrogen) (Reservoir 3). Reservoirs 4 and 5 were left empty. After integration in the EUs, cells destined for the ISS remained for 4 days and 22 h at around 27 °C before they were assembled with control electronics and integrated inside the KIC-SL (Kayser Italia Containers-Single Level) (Fig. 11). Then, for the upload on board the launcher, the EUs were placed inside the BIOKIT transportation container, which is a soft pouch containing phase change materials (PCMbricks) conditioned at +27 °C, until they were inserted into the ESA Kubik facility onboard ISS inside the European Columbus Module where they reached 37 °C in 1 h. At this time, automatized medium change (Reservoir 1) was performed and huMPCs started to be cultured in DM for 7 days. At the end of the experimentation, cell cultures were washed (Reservoir 2) and added to RNA-Later solution (Reservoir 3) to preserve RNAs until analysis. The EUs were placed into the MELFI (Minus Eighty-Degree Laboratory Freezer for ISS) cold stowage asset until landing. For on-ground experiments, EUs were kept in a controlled T environment according to the temperature values and timings registered for the ISS experiments.

### Scanning electron microscopy

Cells were seeded in two 24-well plates (6000 cells/well; 10 coverslips/plate). Five days later, coverslips were washed with PBS and fixed with 2% (w/v) glutaraldehyde (Electron Microscopy Sciences, Hatfield, PA) in PBS for 15 min at RT. Then, coverslips were washed twice with PBS for 5 min at RT. Cells were gradually dehydrated with ethanol series (30–100%) and dried through a graduated series of ethanol:HMDS mixtures (2:1 ethanol:HMDS and 1:2 ethanol:HMDS, respectively), until pure HMDS (Hexamethyldisilazane; Electron Microscopy Sciences). Finally, samples were analyzed with a Zeiss GeminiSEM 500 (Zeiss, Oberkochen, Germany).

### Microarray analysis

Total RNA was extracted with TRI-Reagent (Sigma-Aldrich, Milan, Italy) from huMPCs isolated from the pre-flight astronaut and the age- and sex-matched volunteer subject cultured on the ground or on board the ISS. Quantification and quality control were performed by the CRIBI Biotechnology Center (University of Padua, Padua, Italy) with Nano Drop and Agilent Chip. Gene expression was detected using a Microarray Human Gene Expression platform V3 8X60K Agilent (Agilent Technologies, Santa Clara, CA) after the following steps: RNA quality control (degradation) on Nano Chip Agilent; amplification and total RNA marking using “one-color amplification”; dynamic hybridization on Agilent heater; microarray Agilent Human 8X60K; scanning procedure using DNA Microarray scanner Agilent with 3 μm resolution. Each huMPC sample was cultured in duplicate on board the ISS or on the ground. For each subject, the pooled total RNAs were subjected to microarray analysis (Space vs. Ground) in triplicate. Heatmap was plotted by SRplot (https://www.bioinformatics.com.cn/en), a free online platform for data analysis and visualization^62^. Functional annotation and gene ontology (GO) enrichment analyses were performed with the updated DAVID (*Database for Annotation, Visualization and Integrated Discovery*) 2021 bioinformatics resource (https://david.ncifcrf.gov/home.jsp)^63^ or WebGestalt (WEB-based Gene SeT AnaLysis Toolkit) 2024 (https://www.webgestalt.org/)^64^ (fold change cut-off, 0.7).

### miRNA expression profile

Total RNAs extracted from huMPCs of the pre-flight astronaut and the age- and sex-matched volunteer cultured in duplicate on board the ISS or on the ground underwent retro-transcription and real-time PCR were carried out in triplicate (Space vs. Ground) according to the Applied Biosystems TaqMan miRNA assay kit protocols, as previously reported^61^. The specific miRNA sequence probes used were: hsa-miR-1 (#002222); hsa-miR-206 (#000510); hsa-miR-133a (#002246); hsa-miR-133b (#002247); and hsa-miR-16-5p (#000391), all from Thermo Fisher Scientific (Waltham, MA). MiR-16 was used as an endogenous control. Applied Biosystems Prism 7900HT Sequence Detection System was used with the Sequence Detector Software (SDS version 2.0; Applied Biosystems). The relative quantification of the miRNA targets was carried out using the ΔCt formula, Ct_(miRNA-of-interest)_−Ct_(miR-16)_.

### RNA-Seq transcriptome analysis

For RNA-Seq analysis, RNAs were extracted from VL muscle biopsies of pre- and post-flight astronauts with the PureLink™ RNA Mini Kit (Invitrogen, Carlsbad, CA) following the manufacturer’s instructions. RNA quality and quantity were assessed with the Qubit® RNA HS Assay Kit and a Qubit 3.0 fluorometer (Invitrogen), followed by agarose gel electrophoresis. Libraries were generated using the Ion AmpliSeq™ Transcriptome Human Gene Expression Core Panel and Chef-Ready Kit (Comprehensive evaluation of AmpliSeq transcriptome, a whole transcriptome RNA sequencing methodology) (Thermo Fisher Scientific, Waltham, MA). RNA (10 ng) was reverse-transcribed with SuperScript™ Vilo™ cDNA Synthesis Kit (Thermo Fisher Scientific) before library preparation on the Ion Chef™ instrument (Thermo Fisher Scientific). The resulting cDNA was amplified to prepare barcoded libraries using the Ion Code™ PCR Plate, and the Ion AmpliSeq™ Transcriptome Human Gene Expression Core Panel (Thermo Fisher Scientific), Chef-Ready Kit, according to the manufacturer’s instructions. Barcoded libraries were combined to a final concentration of 100 pM and used to prepare Template-Positive Ion Sphere™ (Thermo Fisher Scientific) Particles to load on Ion 540™ Chips, using the Ion 540™ Kit-Chef (Thermo Fisher Scientific). Sequencing was performed on an Ion S5™ Sequencer with Torrent Suite™ Software v12 (Thermo Fisher Scientific). RNA-Seq analysis of post- vs. pre-flight muscle samples was performed in duplicate. The analyses were performed with 1.5 or 2.0 as fold change cut-off (*P*-value < 0.05) using Transcriptome Analysis Console Software (version 4.0.1), certified for AmpliSeq analysis (Thermo Fisher). Heatmap was plotted by https://www.bioinformatics.com.cn/en. Functional annotation and GO enrichment analyses were performed with DAVID 2021^63^ (fold change cut-off, 2.0) or WebGestalt (WEB-based Gene SeT AnaLysis Toolkit) 2019 (https://www.webgestalt.org/)^64^ (fold change cut-off, 1.5).

### Statistical analysis

The test used for statistical analysis is reported in the legend of each figure. Quantifications of desmin^+^ cells and fusion indexes were performed by two independent operators blinded to treatments. The data are reported as means ± standard deviation of the mean (SD). GraphPad Prism Software, version 9.3.1 (GraphPad Software, La Jolla, CA) was used to assess the significance of the differences in desmin^+^ cells and fusion indexes, and myomiR expressions. *P*-value < 0.05 was considered as statistically significant. Microarray and RNA-Seq analyses were performed by external operators blinded to treatments. Welch’s *t*-test or ANOVA method (eBayes-corrected variance) was used to reveal statistical differences (*P*-value < 0.05) in microarray and RNA-Seq analysis, respectively. EASE Score, a modified Fisher Exact *P*-value, was used to measure the gene enrichment in annotation terms in DAVID, and a *P*-value < 0.05 was considered as statistically significant. In GSEA analysis (WebGestalt), the false discovery rate (FDR) was applied as a multiple testing correction method, and FDR < 0.05 was considered as statistically significant.

## Supplementary information

Supplementary Information

## Data Availability

The datasets generated and analyzed in the current study are not publicly available due to privacy reasons. They are available from the corresponding authors after reasonable request.
