# Supplementary material for: The MyoGravity project to study real microgravity effects on human muscle precursor cells and tissue
Source: NPJ Microgravity. 2024 Oct 3;10:92. doi: 10.1038/s41526-024-00432-1 (PMC11450100; doi:10.1038/s41526-024-00432-1)
Supplement: Supplementary file 1 — Supplementary Information [file 41526_2024_432_MOESM1_ESM.docx]

**SUPPLEMENTARY MATERIAL**


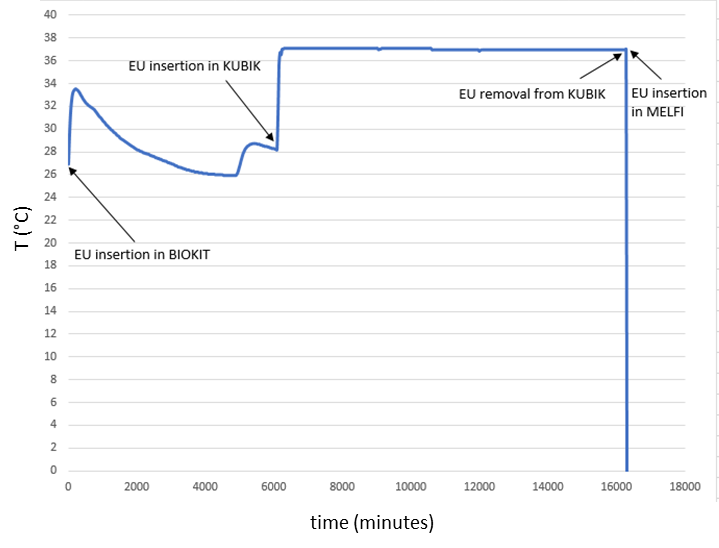


**Figure S1.** Temperature (°C) profile *vs* time (min) from the integration of the experiment units (EUs) at the Kennedy Space Centre facilities to their insertion into the KUBIK incubator on board the ISS, and subsequent insertion into the MELFI cold stowage asset at the end of the experimental procedure. After integration in the EUs, huMPCs remained for 4 days and 16 hrs at around 27.5°C until they were inserted into the KUBIK where they reached 37°C in 1 h. At this time, automatized washing was performed and huMPCs started to be cultured in differentiation medium (DM) for 7 days. Therefore, cell cultures were washed, added with RNA-Later solution, and placed into the MELFI at –80 °C.

**
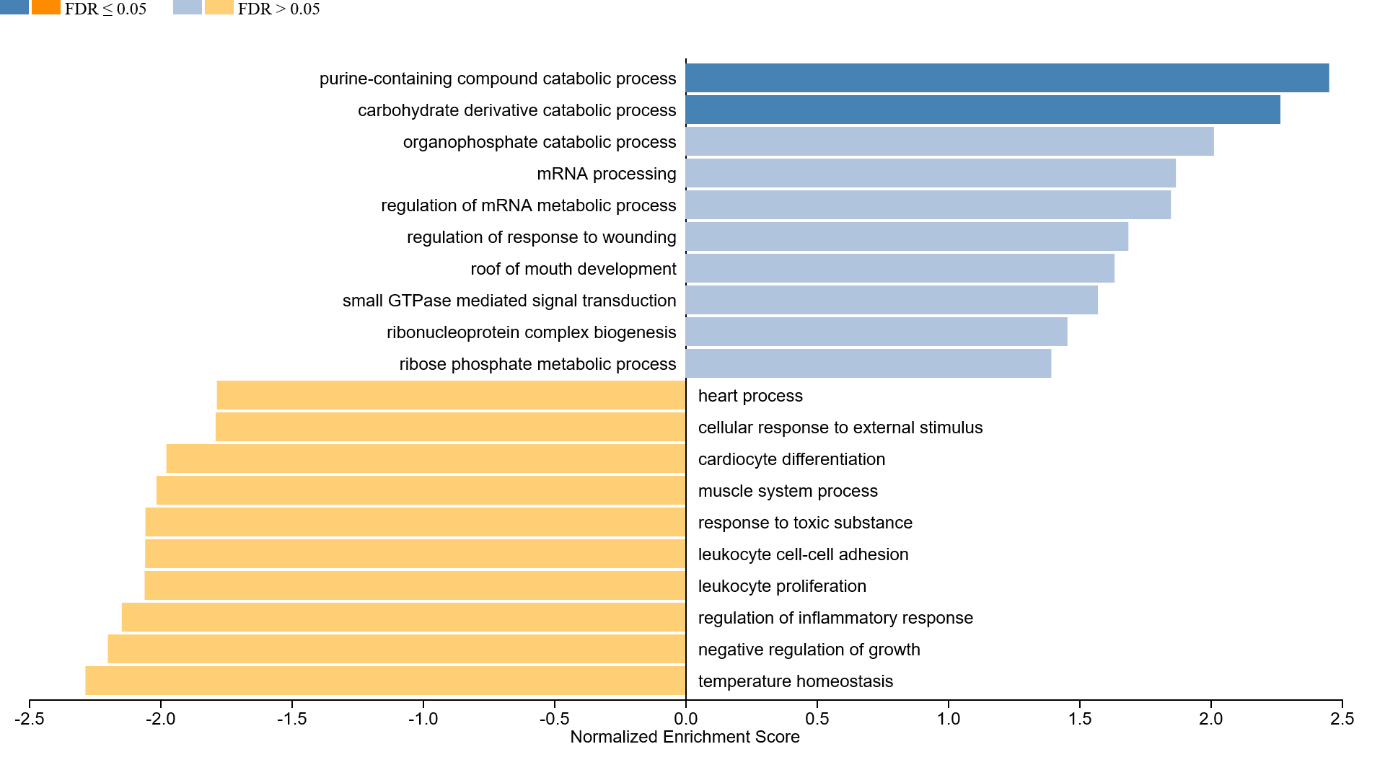
**

**Figure S2.** WebGestalt gene set enrichment analysis (functional category, Biological Process-noRedundant) in Space *vs* Ground huMPCs (log2 fold change cut-off, 0.7). Minimum number of genes in the category, 10. Shown are the resulting significant (FDR<0.05) and not significant (FDR>0.05) categories.

**Table S1.** Microarray analysis. Functional annotation chart of the genes upregulated in common (Space *vs* Ground; log2 fold change cut-off, 0.7) in huMPCs isolated from the pre-flight astronaut and age- and sex-matched volunteer. EASE Score, a modified Fisher Exact *P*-Value, for gene-enrichment analysis. *P* ≤0.05.

| Category | Term | Count | % | P-Value |
| --- | --- | --- | --- | --- |
| INTERPRO | Pleckstrin homology-like domain | 24 | 5.2 | 3.5E-5 |
| GOTERM_BP_DIRECT | synapse assembly | 8 | 1.7 | 4.0E-4 |
| INTERPRO | Pleckstrin homology domain | 16 | 3.5 | 4.0E-4 |
| UP_KW_DOMAIN | Repeat | 136 | 29.6 | 1.2E-3 |
| UP_KW_PTM | Phosphoprotein | 202 | 44.0 | 1.6E-3 |
| INTERPRO | Cadherin, N-terminal | 7 | 1.5 | 1.7E-3 |
| UP_KW_BIOLOGICAL_PROCESS | Ion transport | 26 | 5.7 | 1.9E-3 |
| GOTERM_BP_DIRECT | regulation of small GTPase mediated signal transduction | 9 | 2.0 | 2.0E-3 |
| SMART | PH (Pleckstrin homology domain) | 15 | 3.3 | 2.0E-3 |
| GOTERM_BP_DIRECT | calcium-dependent cell-cell adhesion via plasma membrane cell adhesion molecules | 6 | 1.3 | 2.1E-3 |
| UP_KW_BIOLOGICAL_PROCESS | Cell adhesion | 21 | 4.6 | 2.4E-3 |
| UP_KW_MOLECULAR_FUNCTION | Voltage-gated channel | 10 | 2.2 | 3.2E-3 |
| GOTERM_CC_DIRECT | integral component of membrane | 128 | 27.9 | 3.4E-3 |
| INTERPRO | Band 4.1 domain | 6 | 1.3 | 3.5E-3 |
| INTERPRO | FERM central domain | 6 | 1.3 | 3.5E-3 |
| INTERPRO | Cytochrome P450, E-class, group I, CYP2D-like | 3 | 0.7 | 3.7E-3 |
| INTERPRO | FERM/acyl-CoA-binding protein, 3-helical bundle | 6 | 1.3 | 3.8E-3 |
| GOTERM_BP_DIRECT | regulation of ion transmembrane transport | 9 | 2.0 | 4.2E-3 |
| INTERPRO | Furin-like repeat | 4 | 0.9 | 5.0E-3 |
| GOTERM_BP_DIRECT | cell adhesion | 21 | 4.6 | 5.0E-3 |
| SMART | B41 (Band 4.1 homologues) | 6 | 1.3 | 5.2E-3 |
| GOTERM_BP_DIRECT | regulation of cell migration | 8 | 1.7 | 5.4E-3 |
| UP_KW_DISEASE | Intellectual disability | 30 | 6.5 | 5.6E-3 |
| INTERPRO | Insulin-like growth factor binding protein, N-terminal | 9 | 2.0 | 6.3E-3 |
| GOTERM_CC_DIRECT | membrane | 92 | 20.0 | 6.4E-3 |
| SMART | FU (Furin-like repeats) | 4 | 0.9 | 6.5E-3 |
| INTERPRO | Cadherin conserved site | 8 | 1.7 | 7.1E-3 |
| INTERPRO | Cadherin | 8 | 1.7 | 8.9E-3 |
| GOTERM_BP_DIRECT | chemical synaptic transmission | 12 | 2.6 | 9.8E-3 |
| INTERPRO | Cadherin-like | 8 | 1.7 | 1.0E-2 |
| GOTERM_CC_DIRECT | trans-Golgi network | 10 | 2.2 | 1.1E-2 |
| GOTERM_CC_DIRECT | histone methyltransferase complex | 4 | 0.9 | 1.2E-2 |
| UP_KW_BIOLOGICAL_PROCESS | Transport | 58 | 12.6 | 1.2E-2 |
| GOTERM_BP_DIRECT | cAMP catabolic process | 3 | 0.7 | 1.2E-2 |
| GOTERM_MF_DIRECT | transmembrane transporter activity | 9 | 2.0 | 1.2E-2 |
| SMART | CA (Cadherin repeats) | 8 | 1.7 | 1.3E-2 |
| GOTERM_MF_DIRECT | actin filament binding | 11 | 2.4 | 1.3E-2 |
| UP_KW_LIGAND | Calcium | 34 | 7.4 | 1.4E-2 |
| INTERPRO | Dbl homology (DH) domain | 6 | 1.3 | 1.5E-2 |
| SMART | RhoGEF (Guanine nucleotide exchange factor for Rho/Rac/Cdc42-like GTPases) | 6 | 1.3 | 1.9E-2 |
| INTERPRO | Bromo adjacent homology (BAH) domain | 3 | 0.7 | 1.9E-2 |
| GOTERM_BP_DIRECT | histone H3-K4 methylation | 4 | 0.9 | 1.9E-2 |
| INTERPRO | Ion transport domain | 7 | 1.5 | 2.0E-2 |
| INTERPRO | FERM domain | 5 | 1.1 | 2.0E-2 |
| GOTERM_BP_DIRECT | thyroid hormone transport | 3 | 0.7 | 2.1E-2 |
| SMART | BAH (Bromo adjacent homology domain) | 3 | 0.7 | 2.3E-2 |
| GOTERM_CC_DIRECT | azurophil granule membrane | 5 | 1.1 | 2.5E-2 |
| GOTERM_MF_DIRECT | extracellular matrix binding | 4 | 0.9 | 2.5E-2 |
| GOTERM_CC_DIRECT | integral component of plasma membrane | 39 | 8.5 | 2.6E-2 |
| GOTERM_BP_DIRECT | signal transduction | 35 | 7.6 | 2.6E-2 |
| GOTERM_BP_DIRECT | nervous system development | 15 | 3.3 | 2.7E-2 |
| UP_KW_MOLECULAR_FUNCTION | Repressor | 21 | 4.6 | 2.8E-2 |
| UP_KW_MOLECULAR_FUNCTION | Ion channel | 14 | 3.1 | 3.3E-2 |
| GOTERM_CC_DIRECT | plasma membrane | 119 | 25.9 | 3.3E-2 |
| GOTERM_CC_DIRECT | voltage-gated calcium channel complex | 4 | 0.9 | 3.5E-2 |
| GOTERM_CC_DIRECT | cytoskeleton | 17 | 3.7 | 3.5E-2 |
| GOTERM_BP_DIRECT | establishment of epithelial cell polarity | 3 | 0.7 | 3.6E-2 |
| KEGG_PATHWAY | Taurine and hypotaurine metabolism | 3 | 0.7 | 3.7E-2 |
| INTERPRO | Voltage-dependent potassium channel, four helix bundle domain | 5 | 1.1 | 3.7E-2 |
| GOTERM_BP_DIRECT | positive regulation of nitric oxide metabolic process | 2 | 0.4 | 3.8E-2 |
| GOTERM_BP_DIRECT | negative regulation of relaxation of cardiac muscle | 2 | 0.4 | 3.8E-2 |
| GOTERM_BP_DIRECT | detection of bacterial lipoprotein | 2 | 0.4 | 3.8E-2 |
| INTERPRO | Src homology-3 domain | 10 | 2.2 | 3.8E-2 |
| UP_KW_BIOLOGICAL_PROCESS | Potassium transport | 7 | 1.5 | 3.8E-2 |
| GOTERM_MF_DIRECT | 3',5'-cyclic-AMP phosphodiesterase activity | 3 | 0.7 | 3.9E-2 |
| INTERPRO | Protein of unknown function DUF3827 | 2 | 0.4 | 3.9E-2 |
| INTERPRO | Adenomatous polyposis coli 2 | 2 | 0.4 | 3.9E-2 |
| GOTERM_MF_DIRECT | ATPase activity | 15 | 3.3 | 3.9E-2 |
| INTERPRO | Rho GTPase activation protein | 6 | 1.3 | 4.0E-2 |
| GOTERM_BP_DIRECT | insulin secretion | 4 | 0.9 | 4.0E-2 |
| GOTERM_BP_DIRECT | innervation | 3 | 0.7 | 4.1E-2 |
| GOTERM_BP_DIRECT | regulation of signal transduction by p53 class mediator | 4 | 0.9 | 4.3E-2 |
| GOTERM_BP_DIRECT | negative regulation of canonical Wnt signaling pathway | 8 | 1.7 | 4.4E-2 |
| GOTERM_BP_DIRECT | anion transmembrane transport | 3 | 0.7 | 4.5E-2 |
| GOTERM_BP_DIRECT | heart morphogenesis | 4 | 0.9 | 4.6E-2 |
| GOTERM_MF_DIRECT | amino acid transmembrane transporter activity | 4 | 0.9 | 4.7E-2 |
| GOTERM_MF_DIRECT | calcium channel regulator activity | 4 | 0.9 | 4.7E-2 |
| GOTERM_CC_DIRECT | cell cortex | 8 | 1.7 | 4.7E-2 |
| GOTERM_CC_DIRECT | synapse | 17 | 3.7 | 4.7E-2 |
| GOTERM_BP_DIRECT | angiogenesis | 10 | 2.2 | 4.8E-2 |
| SMART | VWC (von Willebrand factor (vWF) type C domain) | 4 | 0.9 | 4.9E-2 |
| GOTERM_BP_DIRECT | animal organ morphogenesis | 7 | 1.5 | 4.9E-2 |
| INTERPRO | Rho GTPase-activating protein domain | 5 | 1.1 | 4.9E-2 |
| INTERPRO | von Willebrand factor, type C | 4 | 0.9 | 4.9E-2 |
| GOTERM_BP_DIRECT | homophilic cell adhesion via plasma membrane adhesion molecules | 8 | 1.7 | 5.0E-2 |

**Table S2.** Microarray analysis. Functional annotation chart of the genes downregulated in common (Space *vs* Ground; log2 fold change cut-off, 0.7) in huMPCs isolated from the pre-flight astronaut and age- and sex-matched volunteer. EASE Score, a modified Fisher Exact *P*-Value, for gene-enrichment analysis. *P* ≤0.05.

| Category | Term | Count | % | P-Value |
| --- | --- | --- | --- | --- |
| UP_KW_MOLECULAR_FUNCTION | Muscle protein | 13 | 2.8 | 2.9E-9 |
| GOTERM_MF_DIRECT | structural constituent of muscle | 11 | 2.4 | 3.2E-9 |
| GOTERM_BP_DIRECT | sarcomere organization | 9 | 1.9 | 5.3E-7 |
| GOTERM_BP_DIRECT | cardiac muscle contraction | 9 | 1.9 | 7.9E-7 |
| GOTERM_BP_DIRECT | regulation of the force of heart contraction | 6 | 1.3 | 1.0E-5 |
| INTERPRO | Metallothionein, vertebrate, metal binding site | 5 | 1.1 | 3.0E-5 |
| GOTERM_MF_DIRECT | actin binding | 19 | 4.1 | 4.6E-5 |
| INTERPRO | Metallothionein, vertebrate | 5 | 1.1 | 8.6E-5 |
| UP_KW_LIGAND | Metal-thiolate cluster | 5 | 1.1 | 8.9E-5 |
| GOTERM_CC_DIRECT | muscle myosin complex | 5 | 1.1 | 1.1E-4 |
| INTERPRO | Metallothionein domain, vertebrate | 5 | 1.1 | 1.2E-4 |
| INTERPRO | Metallothionein domain | 5 | 1.1 | 1.2E-4 |
| INTERPRO | Metallothionein superfamily, eukaryotic | 5 | 1.1 | 1.2E-4 |
| GOTERM_BP_DIRECT | macrophage chemotaxis | 5 | 1.1 | 1.3E-4 |
| GOTERM_CC_DIRECT | sarcomere | 7 | 1.5 | 1.5E-4 |
| UP_KW_CELLULAR_COMPONENT | Thick filament | 5 | 1.1 | 2.0E-4 |
| GOTERM_BP_DIRECT | muscle contraction | 9 | 1.9 | 2.1E-4 |
| GOTERM_BP_DIRECT | detoxification of copper ion | 5 | 1.1 | 2.2E-4 |
| UP_KW_PTM | Methylation | 38 | 8.2 | 2.5E-4 |
| GOTERM_CC_DIRECT | myofibril | 6 | 1.3 | 2.6E-4 |
| UP_KW_DISEASE | Cardiomyopathy | 10 | 2.2 | 3.6E-4 |
| UP_KW_MOLECULAR_FUNCTION | Myosin | 7 | 1.5 | 4.4E-4 |
| GOTERM_CC_DIRECT | myosin filament | 5 | 1.1 | 4.5E-4 |
| GOTERM_BP_DIRECT | regulation of nitric-oxide synthase activity | 4 | 0.9 | 4.7E-4 |
| GOTERM_CC_DIRECT | Z disc | 10 | 2.2 | 5.3E-4 |
| GOTERM_BP_DIRECT | cellular response to cadmium ion | 6 | 1.3 | 5.4E-4 |
| UP_KW_LIGAND | Cadmium | 4 | 0.9 | 6.3E-4 |
| GOTERM_CC_DIRECT | myosin II complex | 5 | 1.1 | 6.5E-4 |
| KEGG_PATHWAY | Dilated cardiomyopathy | 9 | 1.9 | 7.8E-4 |
| GOTERM_BP_DIRECT | cellular response to zinc ion | 5 | 1.1 | 1.0E-3 |
| UP_KW_MOLECULAR_FUNCTION | Actin-binding | 15 | 3.2 | 1.3E-3 |
| GOTERM_MF_DIRECT | phospholipid transporter activity | 4 | 0.9 | 1.4E-3 |
| GOTERM_BP_DIRECT | cellular response to metal ion | 4 | 0.9 | 1.5E-3 |
| GOTERM_BP_DIRECT | cellular response to copper ion | 5 | 1.1 | 1.6E-3 |
| GOTERM_CC_DIRECT | A band | 4 | 0.9 | 1.7E-3 |
| GOTERM_BP_DIRECT | cardiac muscle hypertrophy in response to stress | 4 | 0.9 | 1.9E-3 |
| GOTERM_BP_DIRECT | adult heart development | 4 | 0.9 | 1.9E-3 |
| KEGG_PATHWAY | Hypertrophic cardiomyopathy | 8 | 1.7 | 2.5E-3 |
| GOTERM_CC_DIRECT | extracellular space | 52 | 11.2 | 2.7E-3 |
| GOTERM_MF_DIRECT | structural constituent of cytoskeleton | 8 | 1.7 | 2.9E-3 |
| KEGG_PATHWAY | Adrenergic signaling in cardiomyocytes | 10 | 2.2 | 3.7E-3 |
| GOTERM_BP_DIRECT | negative regulation of growth | 4 | 0.9 | 4.7E-3 |
| GOTERM_BP_DIRECT | cellular zinc ion homeostasis | 5 | 1.1 | 5.0E-3 |
| UP_KW_MOLECULAR_FUNCTION | Motor protein | 9 | 1.9 | 5.3E-3 |
| GOTERM_CC_DIRECT | extracellular region | 54 | 11.6 | 5.8E-3 |
| GOTERM_CC_DIRECT | actin cytoskeleton | 12 | 2.6 | 6.5E-3 |
| GOTERM_MF_DIRECT | actin filament binding | 11 | 2.4 | 6.9E-3 |
| GOTERM_BP_DIRECT | cardiac muscle cell development | 4 | 0.9 | 8.2E-3 |
| GOTERM_CC_DIRECT | M band | 4 | 0.9 | 8.4E-3 |
| UP_KW_CELLULAR_COMPONENT | Secreted | 54 | 11.6 | 8.6E-3 |
| UP_KW_DOMAIN | LIM domain | 6 | 1.3 | 8.8E-3 |
| GOTERM_CC_DIRECT | cytoskeleton | 18 | 3.9 | 9.4E-3 |
| GOTERM_MF_DIRECT | kinase binding | 7 | 1.5 | 9.9E-3 |
| GOTERM_BP_DIRECT | ventricular cardiac muscle tissue morphogenesis | 4 | 0.9 | 1.0E-2 |
| UP_KW_LIGAND | Copper | 6 | 1.3 | 1.1E-2 |
| GOTERM_CC_DIRECT | lipid particle | 7 | 1.5 | 1.1E-2 |
| GOTERM_CC_DIRECT | myosin complex | 5 | 1.1 | 1.1E-2 |
| INTERPRO | Zinc finger, LIM-type | 6 | 1.3 | 1.1E-2 |
| GOTERM_BP_DIRECT | skeletal muscle contraction | 4 | 0.9 | 1.2E-2 |
| SMART | LIM (Zinc-binding domain present in Lin-11, Isl-1, Mec-3) | 6 | 1.3 | 1.3E-2 |
| UP_KW_PTM | Phosphoprotein | 181 | 38.9 | 1.3E-2 |
| GOTERM_BP_DIRECT | regulation of blood pressure | 6 | 1.3 | 1.3E-2 |
| GOTERM_BP_DIRECT | positive regulation of gene expression | 18 | 3.9 | 1.4E-2 |
| GOTERM_BP_DIRECT | signal transduction | 35 | 7.5 | 1.5E-2 |
| GOTERM_MF_DIRECT | actin monomer binding | 4 | 0.9 | 1.6E-2 |
| GOTERM_BP_DIRECT | apoptotic process | 20 | 4.3 | 1.6E-2 |
| UP_KW_MOLECULAR_FUNCTION | Elongation factor | 4 | 0.9 | 1.7E-2 |
| GOTERM_BP_DIRECT | neutrophil chemotaxis | 6 | 1.3 | 1.7E-2 |
| GOTERM_MF_DIRECT | cyclin-dependent protein serine/threonine kinase regulator activity | 4 | 0.9 | 1.7E-2 |
| GOTERM_MF_DIRECT | translation elongation factor activity | 4 | 0.9 | 1.7E-2 |
| GOTERM_BP_DIRECT | cardiac myofibril assembly | 3 | 0.6 | 2.0E-2 |
| GOTERM_BP_DIRECT | cardiac muscle hypertrophy | 3 | 0.6 | 2.0E-2 |
| UP_KW_MOLECULAR_FUNCTION | Protein phosphatase inhibitor | 4 | 0.9 | 2.0E-2 |
| GOTERM_MF_DIRECT | protein serine/threonine phosphatase inhibitor activity | 3 | 0.6 | 2.2E-2 |
| GOTERM_CC_DIRECT | RNA polymerase II transcription factor complex | 7 | 1.5 | 2.2E-2 |
| INTERPRO | Cyclin, N-terminal | 4 | 0.9 | 2.4E-2 |
| INTERPRO | Cytokine, IL-1-like | 4 | 0.9 | 2.4E-2 |
| GOTERM_MF_DIRECT | lipid binding | 9 | 1.9 | 2.5E-2 |
| GOTERM_CC_DIRECT | cell periphery | 5 | 1.1 | 2.5E-2 |
| GOTERM_BP_DIRECT | regulation of heart rate | 4 | 0.9 | 2.6E-2 |
| INTERPRO | EF-hand domain | 10 | 2.2 | 2.6E-2 |
| GOTERM_BP_DIRECT | regulation of muscle contraction | 3 | 0.6 | 2.6E-2 |
| INTERPRO | Intermediate filament protein, conserved site | 5 | 1.1 | 2.7E-2 |
| INTERPRO | Myosin S1 fragment, N-terminal | 3 | 0.6 | 2.9E-2 |
| GOTERM_MF_DIRECT | interleukin-1 receptor binding | 3 | 0.6 | 2.9E-2 |
| INTERPRO | EF-hand-like domain | 11 | 2.4 | 2.9E-2 |
| GOTERM_BP_DIRECT | lipid transport | 6 | 1.3 | 2.9E-2 |
| GOTERM_BP_DIRECT | negative regulation of calcineurin-NFAT signaling cascade | 3 | 0.6 | 3.0E-2 |
| GOTERM_BP_DIRECT | response to pain | 3 | 0.6 | 3.0E-2 |
| GOTERM_BP_DIRECT | transcription from RNA polymerase II promoter | 10 | 2.2 | 3.2E-2 |
| INTERPRO | DNA repair protein XRCC4, C-terminal | 3 | 0.6 | 3.2E-2 |
| GOTERM_CC_DIRECT | ruffle membrane | 6 | 1.3 | 3.3E-2 |
| KEGG_PATHWAY | Cardiac muscle contraction | 6 | 1.3 | 3.3E-2 |
| GOTERM_BP_DIRECT | replicative senescence | 3 | 0.6 | 3.4E-2 |
| GOTERM_BP_DIRECT | striated muscle contraction | 3 | 0.6 | 3.4E-2 |
| GOTERM_BP_DIRECT | muscle filament sliding | 3 | 0.6 | 3.4E-2 |
| GOTERM_BP_DIRECT | regulation of phosphorylation | 3 | 0.6 | 3.4E-2 |
| GOTERM_BP_DIRECT | positive regulation of JNK cascade | 6 | 1.3 | 3.4E-2 |
| INTERPRO | Cyclin-like | 4 | 0.9 | 3.4E-2 |
| GOTERM_BP_DIRECT | acute-phase response | 4 | 0.9 | 3.4E-2 |
| GOTERM_BP_DIRECT | positive regulation of T cell proliferation | 5 | 1.1 | 3.4E-2 |
| INTERPRO | Interleukin-1 propeptide | 2 | 0.4 | 3.5E-2 |
| GOTERM_MF_DIRECT | myosin II heavy chain binding | 2 | 0.4 | 3.5E-2 |
| SMART | CYCLIN (domain present in cyclins, TFIIB and Retinoblastoma) | 4 | 0.9 | 3.6E-2 |
| GOTERM_BP_DIRECT | negative regulation of odontogenesis of dentin-containing tooth | 2 | 0.4 | 3.6E-2 |
| GOTERM_BP_DIRECT | epithelial cell differentiation | 6 | 1.3 | 3.7E-2 |
| UP_KW_PTM | Oxidation | 4 | 0.9 | 3.7E-2 |
| UP_KW_CELLULAR_COMPONENT | Cytoplasm | 116 | 24.9 | 3.8E-2 |
| GOTERM_BP_DIRECT | DNA damage response, signal transduction by p53 class mediator | 3 | 0.6 | 3.8E-2 |
| GOTERM_CC_DIRECT | stress fiber | 5 | 1.1 | 3.8E-2 |
| UP_KW_MOLECULAR_FUNCTION | Pyrogen | 2 | 0.4 | 3.8E-2 |
| GOTERM_BP_DIRECT | cellular response to reactive oxygen species | 4 | 0.9 | 3.9E-2 |
| GOTERM_CC_DIRECT | costamere | 3 | 0.6 | 3.9E-2 |
| GOTERM_CC_DIRECT | CENP-A containing nucleosome | 3 | 0.6 | 3.9E-2 |
| UP_KW_BIOLOGICAL_PROCESS | Apoptosis | 15 | 3.2 | 4.0E-2 |
| GOTERM_CC_DIRECT | cyclin-dependent protein kinase holoenzyme complex | 4 | 0.9 | 4.0E-2 |
| GOTERM_MF_DIRECT | apolipoprotein binding | 3 | 0.6 | 4.0E-2 |
| GOTERM_BP_DIRECT | cholesterol homeostasis | 6 | 1.3 | 4.1E-2 |
| UP_KW_BIOLOGICAL_PROCESS | Myogenesis | 4 | 0.9 | 4.1E-2 |
| GOTERM_CC_DIRECT | focal adhesion | 14 | 3.0 | 4.1E-2 |
| GOTERM_CC_DIRECT | adherens junction | 8 | 1.7 | 4.1E-2 |
| GOTERM_BP_DIRECT | positive regulation of protein phosphorylation | 9 | 1.9 | 4.2E-2 |
| GOTERM_BP_DIRECT | negative regulation of cell proliferation | 15 | 3.2 | 4.2E-2 |
| GOTERM_BP_DIRECT | positive regulation of vascular endothelial growth factor receptor signaling pathway | 3 | 0.6 | 4.2E-2 |
| GOTERM_BP_DIRECT | protein localization to CENP-A containing chromatin | 3 | 0.6 | 4.2E-2 |
| INTERPRO | Myosin, N-terminal, SH3-like | 3 | 0.6 | 4.4E-2 |
| INTERPRO | Myosin tail | 3 | 0.6 | 4.4E-2 |
| UP_KW_MOLECULAR_FUNCTION | Mitogen | 4 | 0.9 | 4.5E-2 |
| GOTERM_CC_DIRECT | cytosolic ribosome | 5 | 1.1 | 4.5E-2 |
| GOTERM_BP_DIRECT | response to vitamin A | 3 | 0.6 | 4.6E-2 |
| KEGG_PATHWAY | Regulation of actin cytoskeleton | 10 | 2.2 | 4.7E-2 |
| UP_KW_BIOLOGICAL_PROCESS | Lipid transport | 7 | 1.5 | 4.7E-2 |
| UP_KW_MOLECULAR_FUNCTION | Cyclin | 4 | 0.9 | 4.8E-2 |
| KEGG_PATHWAY | Staphylococcus aureus infection | 6 | 1.3 | 4.8E-2 |

**Table S3.** Microarray analysis. Most significant terms of functional annotation chart among the genes upregulated in common (Space *vs* Ground; log2 fold change cut-off, 0.7) in huMPCs isolated from the pre-flight astronaut and age- and sex-matched volunteer.

| Term: Pleckstrin homology-like domain | |
| --- | --- |
| GENE ID | **GENE NAME** |
| 23150 | FERM domain containing 4B(FRMD4B) |
| 84978 | FERM domain containing 5(FRMD5) |
| 83786 | FERM domain containing 8(FRMD8) |
| 55612 | FERM domain containing kindlin 1(FERMT1) |
| 889 | KRIT1 ankyrin repeat containing(KRIT1) |
| 23263 | MCF.2 cell line derived transforming sequence like(MCF2L) |
| 4168 | MCF.2 cell line derived transforming sequence(MCF2) |
| 23239 | PH domain and leucine rich repeat protein phosphatase 1(PHLPP1) |
| 9938 | Rho GTPase activating protein 25(ARHGAP25) |
| 201176 | Rho GTPase activating protein 27(ARHGAP27) |
| 64333 | Rho GTPase activating protein 9(ARHGAP9) |
| 23370 | Rho/Rac guanine nucleotide exchange factor 18(ARHGEF18) |
| 54885 | TBC1 domain family member 8B(TBC1D8B) |
| 23294 | ankyrin repeat and sterile alpha motif domain containing 1A(ANKS1A) |
| 285489 | docking protein 7(DOK7) |
| 3667 | insulin receptor substrate 1(IRS1) |
| 4651 | myosin X (MYO10) |
| 8776 | myotubularin related protein 1(MTMR1) |
| 84033 | obscurin, cytoskeletal calmodulin and titin-interacting RhoGEF(OBSCN) |
| 5334 | phospholipase C like 1 (inactive)(PLCL1) |
| 26499 | pleckstrin 2(PLEK2) |
| 9842 | pleckstrin homology and RUN domain containing M1(PLEKHM1) |
| 55671 | protein phosphatase 4 regulatory subunit 3A(PPP4R3A) |
| 221178 | spermatogenesis associated 13(SPATA13) |
|  | |
| Term: Synapse assembly | |
| GENE ID | **GENE NAME** |
| 5457 | POU class 4 homeobox 1(POU4F1) |
| 28988 | drebrin like(DBNL) |
| 56133 | protocadherin beta 2(PCDHB2) |
| 26167 | protocadherin beta 5(PCDHB5) |
| 56127 | protocadherin beta 9(PCDHB9) |
| 56126 | protocadherin beta 10(PCDHB10) |
| 56122 | protocadherin beta 14(PCDHB14) |
| 57717 | protocadherin beta 16(PCDHB16) |
|  | |
| Term: Cadherin, N-terminal | |
| GENE ID | **GENE NAME** |
| 56133 | protocadherin beta 2(PCDHB2) |
| 26167 | protocadherin beta 5(PCDHB5) |
| 56127 | protocadherin beta 9(PCDHB9) |
| 56126 | protocadherin beta 10(PCDHB10) |
| 56124 | protocadherin beta 12(PCDHB12) |
| 56122 | protocadherin beta 14(PCDHB14) |
| 57717 | protocadherin beta 16(PCDHB16) |
|  | |
| Term: Ion transport | |
| GENE ID | **GENE NAME** |
| 11194 | ATP binding cassette subfamily B member 8(ABCB8) |
| 783 | calcium voltage-gated channel auxiliary subunit beta 2(CACNB2) |
| 117155 | cation channel sperm associated 2(CATSPER2) |
| 347732 | cation channel sperm associated 3(CATSPER3) |
| 1184 | chloride voltage-gated channel 5(CLCN5) |
| 1185 | chloride voltage-gated channel 6(CLCN6) |
| 1144 | cholinergic receptor nicotinic delta subunit(CHRND) |
| 1146 | cholinergic receptor nicotinic gamma subunit(CHRNG) |
| 1364 | claudin 4(CLDN4) |
| 57657 | hyperpolarization activated cyclic nucleotide gated potassium channel 3(HCN3) |
| 3954 | leucine zipper and EF-hand containing transmembrane protein 1(LETM1) |
| 284252 | potassium channel tetramerization domain containing 1(KCTD1) |
| 3772 | potassium inwardly rectifying channel subfamily J member 15(KCNJ15) |
| 30818 | potassium voltage-gated channel interacting protein 3(KCNIP3) |
| 3746 | potassium voltage-gated channel subfamily C member 1(KCNC1) |
| 3748 | potassium voltage-gated channel subfamily C member 3(KCNC3) |
| 9132 | potassium voltage-gated channel subfamily Q member 4(KCNQ4) |
| 6329 | sodium voltage-gated channel alpha subunit 4(SCN4A) |
| 9058 | solute carrier family 13 member 2(SLC13A2) |
| 26266 | solute carrier family 13 member 4(SLC13A4) |
| 55676 | solute carrier family 30 member 6(SLC30A6) |
| 91252 | solute carrier family 39 member 13(SLC39A13) |
| 283375 | solute carrier family 39 member 5(SLC39A5) |
| 57835 | solute carrier family 4 member 5(SLC4A5) |
| 54946 | solute carrier family 41 member 3(SLC41A3) |
| 57620 | stromal interaction molecule 2(STIM2) |

**Table S4.** Microarray. Genes of the significant upregulated categories emerged by WebGestalt gene set enrichment analysis (functional category, Biological Process-noRedundant) in Space *vs* Ground huMPCs (log2 fold change cut-off, 0.7).

| Category: Purine-containing compound catabolic process | |
| --- | --- |
| GENE ID | **GENE NAME** |
| [8876](https://www.ncbi.nlm.nih.gov/gene/?term=8876) | vanin 1(VNN1) |
| [221937](https://www.ncbi.nlm.nih.gov/gene/?term=221937) | forkhead box K1(FOXK1) |
| [55753](https://www.ncbi.nlm.nih.gov/gene/?term=55753) | oxoglutarate dehydrogenase L(OGDHL) |
| [8801](https://www.ncbi.nlm.nih.gov/gene/?term=8801) | succinate-CoA ligase GDP-forming subunit beta(SUCLG2) |
| [5142](https://www.ncbi.nlm.nih.gov/gene/?term=5142) | phosphodiesterase 4B(PDE4B) |
| [283927](https://www.ncbi.nlm.nih.gov/gene/?term=283927) | nudix hydrolase 7(NUDT7) |
| [5208](https://www.ncbi.nlm.nih.gov/gene/?term=5208) | 6-phosphofructo-2-kinase/fructose-2,6-biphosphatase 2(PFKFB2) |
| [2033](https://www.ncbi.nlm.nih.gov/gene/?term=2033) | E1A binding protein p300(EP300) |
| [5144](https://www.ncbi.nlm.nih.gov/gene/?term=5144) | phosphodiesterase 4D(PDE4D) |
| 27115 | phosphodiesterase 7B(PDE7B) |
|  | |
| Category: Carbohydrate derivative catabolic process | |
| GENE ID | **GENE NAME** |
| 8876 | vanin 1(VNN1) |
| 10855 | Heparanase(HPSE) |
| 221937 | forkhead box K1(FOXK1) |
| 55753 | oxoglutarate dehydrogenase L(OGDHL) |
| 57379 | activation induced cytidine deaminase(AICDA) |
| 8801 | succinate-CoA ligase GDP-forming subunit beta(SUCLG2) |
| 129530 | lysozyme g1(LYG1) |
| 5142 | phosphodiesterase 4B(PDE4B) |
| 283927 | nudix hydrolase 7(NUDT7) |
| 5208 | 6-phosphofructo-2-kinase/fructose-2,6-biphosphatase 2(PFKFB2) |
| 2033 | E1A binding protein p300(EP300) |
| 5144 | phosphodiesterase 4D(PDE4D) |
| 27115 | phosphodiesterase 7B(PDE7B) |
| 81602 | cytidine and dCMP deaminase domain containing 1(CDADC1) |

**Table S5.** Microarray analysis. Functional annotation chart of the genes upregulated (Space *vs* Ground; log2 fold change cut-off, 1.5) in common in huMPCs isolated from the pre-flight astronaut and age- and sex-matched volunteer. EASE Score, a modified Fisher Exact *P*-Value, for gene-enrichment analysis. *P* ≤0.05.

| Category | Term | Upregulated genes | % | P-Value |
| --- | --- | --- | --- | --- |
| GOTERM_MF_DIRECT | Channel activity | aquaporin 3 (Gill blood group)(AQP3)  cholinergic receptor nicotinic gamma subunit(CHRNG) | 18.2 | 1.1E-2 |
| GOTERM_BP_DIRECT | Odontogenesis | Sp6 transcription factor(SP6)  aquaporin 3 (Gill blood group) (AQP3) | 18.2 | 1.6E-2 |
| UP_KW_CELLULAR_COMPONENT | Cell membrane | EPH receptor B2(EPHB2)  adhesion G protein-coupled receptor E2(ADGRE2)  aquaporin 3 (Gill blood group)(AQP3)  cholinergic receptor nicotinic gamma subunit(CHRNG)  otoferlin(OTOF) | 45.5 | 4.7E-2 |
| INTERPRO | Insulin-like growth factor binding protein, N-terminal | EPH receptor B2(EPHB2)  adhesion G protein-coupled receptor E2(ADGRE2) | 18.2 | 5.7E-2 |
| GOTERM_BP_DIRECT | Adenylate cyclase-activating G-protein coupled receptor signaling pathway | adhesion G protein-coupled receptor E2(ADGRE2)  parathyroid hormone like hormone(PTHLH) | 18.2 | 6.0E-2 |
| UP_KW_CELLULAR_COMPONENT | Cell projection | EPH receptor B2(EPHB2)  adhesion G protein-coupled receptor E2(ADGRE2)  otoferlin(OTOF) | 27.3 | 7.7E-2 |
| GOTERM_CC_DIRECT | Postsynaptic membrane | EPH receptor B2(EPHB2)  cholinergic receptor nicotinic gamma subunit(CHRNG) | 18.2 | 8.3E-2 |

**Table S6.** Microarray analysis. Functional annotation chart of the genes downregulated (Space *vs* Ground; log2 fold change cut-off, 1.5) in common in huMPCs isolated from the pre-flight astronaut and age- and sex-matched volunteer. EASE Score, a modified Fisher Exact *P*-Value, for gene-enrichment analysis. *P* ≤0.05.

| Category | Term | Downregulated genes | % | [P-Value](https://david.ncifcrf.gov/chartReport.jsp?d-16544-s=7&currentList=0&d-16544-o=1&d-16544-p=1&annot=59%2C87%2C88%2C30%2C38%2C46%2C3%2C5%2C55%2C53%2C70%2C79) |
| --- | --- | --- | --- | --- |
| GOTERM_MF_DIRECT | receptor binding | fibrinogen C domain containing 1(FIBCD1)  natriuretic peptide A(NPPA)  neuromedin U(NMU) | 7.5 | 9.4E-2 |
| GOTERM_BP_DIRECT | inflammatory response | cysteine and glycine rich protein 3(CSRP3)  interleukin 1 alpha(IL1A)  phospholipase A2 group IIA(PLA2G2A) | 7.5 | 8.6E-2 |
| GOTERM_MF_DIRECT | transcriptional repressor activity, RNA polymerase II transcription regulatory region sequence-specific binding | E2F transcription factor 7(E2F7)  activating transcription factor 3(ATF3)  coiled-coil and C2 domain containing 1A(CC2D1A) | 7.5 | 6.7E-2 |
| GOTERM_BP_DIRECT | positive regulation of transcription from RNA polymerase II promoter | E2F transcription factor 7(E2F7)  activating transcription factor 3(ATF3)  cysteine and glycine rich protein 3(CSRP3)  interleukin 1 alpha(IL1A)  transcription factor 15(TCF15) | 12.5 | 5.1E-2 |
| GOTERM_BP_DIRECT | cardiac muscle contraction | cysteine and glycine rich protein 3(CSRP3)  myosin light chain 3(MYL3) | 5.0 | 4.9E-2 |
| GOTERM_BP_DIRECT | sarcomere organization | cysteine and glycine rich protein 3(CSRP3)  keratin 19(KRT19) | 5.0 | 4.6E-2 |
| GOTERM_CC_DIRECT | extracellular region | defensin beta (DEFB103B)  eukaryotic translation elongation factor 1 alpha 1(EEF1A1)  interleukin 1 alpha(IL1A)  natriuretic peptide A(NPPA)  neuromedin U(NMU)  perilipin 2(PLIN2)  phospholipase A2 group IIA(PLA2G2A) | 17.5 | 3.8E-2 |
| GOTERM_CC_DIRECT | nucleus | E2F transcription factor 7(E2F7)  MAP3K7 C-terminal like(MAP3K7CL)  activating transcription factor 3(ATF3)  coiled-coil and C2 domain containing 1A(CC2D1A)  cysteine and glycine rich protein 3(CSRP3)  eukaryotic translation elongation factor 1 alpha 1(EEF1A1)  fatty acid binding protein 3(FABP3)  interleukin 1 alpha(IL1A)  metallothionein 1G(MT1G)  musculoskeletal, embryonic nuclear protein 1(MUSTN1)  natriuretic peptide A(NPPA)  perilipin 2(PLIN2)  transcription factor 15(TCF15) | 32.5 | 3.5E-2 |
| GOTERM_CC_DIRECT | extracellular space | defensin beta 103B(DEFB103B)  eukaryotic translation elongation factor 1 alpha 1(EEF1A1)  fatty acid binding protein 3(FABP3)  fibrinogen C domain containing 1(FIBCD1)  interleukin 1 alpha(IL1A)  natriuretic peptide A(NPPA)  phospholipase A2 group IIA(PLA2G2A) | 17.5 | 2.6E-2 |
| GOTERM_BP_DIRECT | positive regulation of I-kappaB kinase/NF-kappaB signaling | ATPase secretory pathway Ca2+ transporting 1(ATP2C1)  coiled-coil and C2 domain containing 1A(CC2D1A)  interleukin 1 alpha(IL1A) | 7.5 | 2.3E-2 |
| GOTERM_BP_DIRECT | regulation of the force of heart contraction | cysteine and glycine rich protein 3(CSRP3)  myosin light chain 3(MYL3) | 5.0 | 2.0E-2 |
| GOTERM_BP_DIRECT | long-chain fatty acid transport | fatty acid binding protein 3(FABP3)  perilipin 2(PLIN2) | 5.0 | 1.9E-2 |
| GOTERM_CC_DIRECT | RNA polymerase II transcription factor complex | E2F transcription factor 7(E2F7)  activating transcription factor 3(ATF3)  transcription factor 15(TCF15) | 7.5 | 9.7E-3 |
| UP_KW_DISEASE | Cardiomyopathy | cysteine and glycine rich protein 3(CSRP3)  myosin light chain 3(MYL3)  natriuretic peptide A(NPPA) | 7.5 | 4.4E-3 |
| GOTERM_MF_DIRECT | structural constituent of muscle | cysteine and glycine rich protein 3(CSRP3)  keratin 19(KRT19)  myosin light chain 3(MYL3) | 7.5 | 1.3E-3 |

**Table S7.** Characteristics and functions of the myomiRs investigated in the study.

| **miRNA**  **(myomiR)** | **Locations**  **miRNA transcript**  **(chromosome band)** | **Host gene** | **Tissue specificity** | **Functional summaries** |
| --- | --- | --- | --- | --- |
| miR-1-3p | hsa-mir-1-1 (20q13.33) hsa-mir-1-2 (18q11.2) | MIR1-1HG (mRNA) MIR133A1HG (lncRNA) | Heart, skeletal muscle | Prevents myoblast proliferation and promotes myoblast differentiation. Reduced in muscle atrophy conditions. |
| miR-133a-3p | hsa-mir-133a-1 (18q11.2) hsa-mir-133a-2 (20q13.33) | MIR133A1HG (lncRNA) MIR1-1HG (mRNA) | Heart, skeletal muscle | Inhibits myoblast proliferation and promotes myoblast differentiation. Reduced in muscle atrophy conditions. |
| miR-133b | hsa-mir-133b (6p12.2) | MIR133BHG  Or LINCMD1 (lncRNA) | Heart, skeletal muscle | Functions coincide with those of miR133a. |
| miR-206 | hsa-mir-206  (6p12.2) |  | Skeletal muscle (Type I) | Involved in myogenesis. Enhances myoblast differentiation and prevents myoblast proliferation. Implicated in muscle atrophy and hypertrophy. |

**Table S8.** RNA-Seq analysis. Functional annotation chart of upregulated genes (Post- *vs* Pre-flight; fold change cut-off, 2.0) in the astronaut’s muscle tissue. EASE Score, a modified Fisher Exact P-Value, for gene-enrichment analysis. *P* ≤0.01.

| [Category](https://david.ncifcrf.gov/chartReport.jsp?d-16544-s=1&currentList=0&d-16544-o=2&d-16544-p=1&annot=59%2C12%2C87%2C88%2C30%2C38%2C46%2C3%2C5%2C55%2C53%2C70%2C79) | [Term](https://david.ncifcrf.gov/chartReport.jsp?d-16544-s=2&currentList=0&d-16544-o=2&d-16544-p=1&annot=59%2C12%2C87%2C88%2C30%2C38%2C46%2C3%2C5%2C55%2C53%2C70%2C79) | [Count](https://david.ncifcrf.gov/chartReport.jsp?d-16544-s=5&currentList=0&d-16544-o=1&d-16544-p=1&annot=59%2C12%2C87%2C88%2C30%2C38%2C46%2C3%2C5%2C55%2C53%2C70%2C79) | [%](https://david.ncifcrf.gov/chartReport.jsp?d-16544-s=6&currentList=0&d-16544-o=1&d-16544-p=1&annot=59%2C12%2C87%2C88%2C30%2C38%2C46%2C3%2C5%2C55%2C53%2C70%2C79) | [P-Value](https://david.ncifcrf.gov/chartReport.jsp?d-16544-s=7&currentList=0&d-16544-o=1&d-16544-p=1&annot=59%2C12%2C87%2C88%2C30%2C38%2C46%2C3%2C5%2C55%2C53%2C70%2C79) |
| --- | --- | --- | --- | --- |
| UP_KW_CELLULAR_COMPONENT | Extracellular matrix | 51 | 5.1 | 1.6E-16 |
| GOTERM_CC_DIRECT | extracellular matrix | 45 | 4.5 | 3.2E-14 |
| GOTERM_BP_DIRECT | cell adhesion | 68 | 6.7 | 6.1E-13 |
| GOTERM_MF_DIRECT | integrin binding | 34 | 3.4 | 6.6E-13 |
| UP_KW_BIOLOGICAL_PROCESS | Cell adhesion | 62 | 6.1 | 5.3E-12 |
| GOTERM_MF_DIRECT | extracellular matrix structural constituent | 30 | 3.0 | 1.7E-11 |
| UP_KW_DOMAIN | Repeat | 339 | 33.6 | 3.1E-10 |
| GOTERM_BP_DIRECT | extracellular matrix organization | 32 | 3.2 | 4.2E-10 |
| GOTERM_MF_DIRECT | actin filament binding | 36 | 3.6 | 6.2E-10 |
| GOTERM_CC_DIRECT | focal adhesion | 51 | 5.1 | 8.1E-10 |
| UP_KW_MOLECULAR_FUNCTION | Actin-binding | 42 | 4.2 | 2.9E-9 |
| GOTERM_BP_DIRECT | collagen fibril organization | 18 | 1.8 | 4.3E-8 |
| INTERPRO | Thrombospondin, type 1 repeat | 17 | 1.7 | 6.3E-8 |
| GOTERM_MF_DIRECT | collagen binding | 17 | 1.7 | 8.1E-8 |
| KEGG_PATHWAY | Focal adhesion | 32 | 3.2 | 1.3E-7 |
| GOTERM_CC_DIRECT | myosin filament | 10 | 1.0 | 1.4E-7 |
| INTERPRO | EGF-like calcium-binding | 23 | 2.3 | 1.8E-7 |
| GOTERM_MF_DIRECT | calcium ion binding | 70 | 6.9 | 1.8E-7 |
| KEGG_PATHWAY | ECM-receptor interaction | 20 | 2.0 | 1.9E-7 |
| GOTERM_CC_DIRECT | plasma membrane | 311 | 30.8 | 2.6E-7 |
| INTERPRO | Collagen triple helix repeat | 18 | 1.8 | 3.4E-7 |
| GOTERM_CC_DIRECT | endoplasmic reticulum lumen | 36 | 3.6 | 5.2E-7 |
| GOTERM_MF_DIRECT | actin binding | 40 | 4.0 | 5.5E-7 |
| SMART | TSP1 (Thrombospondin type 1 repeats) | 17 | 1.7 | 6.7E-7 |
| GOTERM_CC_DIRECT | extracellular region | 144 | 14.3 | 7.3E-7 |
| GOTERM_CC_DIRECT | actin cytoskeleton | 32 | 3.2 | 8.7E-7 |
| GOTERM_CC_DIRECT | basement membrane | 18 | 1.8 | 1.2E-6 |
| INTERPRO | Myosin, N-terminal, SH3-like | 9 | 0.9 | 1.3E-6 |
| INTERPRO | EGF-like calcium-binding, conserved site | 19 | 1.9 | 1.8E-6 |
| GOTERM_BP_DIRECT | cell-cell adhesion | 26 | 2.6 | 3.4E-6 |
| GOTERM_MF_DIRECT | ATP binding | 114 | 11.3 | 3.4E-6 |
| GOTERM_CC_DIRECT | collagen trimer | 17 | 1.7 | 3.5E-6 |
| UP_KW_DOMAIN | EGF-like domain | 32 | 3.2 | 3.7E-6 |
| GOTERM_BP_DIRECT | negative regulation of cell proliferation | 45 | 4.5 | 3.8E-6 |
| UP_KW_MOLECULAR_FUNCTION | Muscle protein | 15 | 1.5 | 4.3E-6 |
| SMART | EGF_CA (Calcium-binding EGF-like domain) | 23 | 2.3 | 4.3E-6 |
| GOTERM_CC_DIRECT | myosin II complex | 9 | 0.9 | 4.7E-6 |
| GOTERM_MF_DIRECT | microfilament motor activity | 11 | 1.1 | 5.1E-6 |
| INTERPRO | Epidermal growth factor-like domain | 30 | 3.0 | 6.0E-6 |
| UP_KW_DOMAIN | Collagen | 18 | 1.8 | 7.2E-6 |
| UP_KW_LIGAND | Calcium | 88 | 8.7 | 8.7E-6 |
| GOTERM_CC_DIRECT | cytosol | 310 | 30.7 | 9.5E-6 |
| KEGG_PATHWAY | Protein digestion and absorption | 19 | 1.9 | 1.0E-5 |
| INTERPRO | Myosin head, motor domain | 11 | 1.1 | 1.4E-5 |
| KEGG_PATHWAY | Dilated cardiomyopathy | 18 | 1.8 | 1.5E-5 |
| INTERPRO | Myosin tail | 8 | 0.8 | 1.7E-5 |
| INTERPRO | Integrin alpha beta-propellor | 8 | 0.8 | 1.7E-5 |
| GOTERM_MF_DIRECT | extracellular matrix structural constituent conferring tensile strength | 11 | 1.1 | 1.8E-5 |
| GOTERM_BP_DIRECT | actin filament organization | 20 | 2.0 | 2.1E-5 |
| UP_KW_MOLECULAR_FUNCTION | Calmodulin-binding | 23 | 2.3 | 2.3E-5 |
| INTERPRO | EGF-type aspartate/asparagine hydroxylation site | 17 | 1.7 | 2.4E-5 |
| GOTERM_MF_DIRECT | heparin binding | 23 | 2.3 | 3.6E-5 |
| INTERPRO | FG-GAP repeat | 8 | 0.8 | 3.7E-5 |
| GOTERM_BP_DIRECT | positive regulation of fibroblast proliferation | 12 | 1.2 | 3.7E-5 |
| INTERPRO | Myosin S1 fragment, N-terminal | 7 | 0.7 | 4.2E-5 |
| GOTERM_BP_DIRECT | actin cytoskeleton organization | 23 | 2.3 | 4.6E-5 |
| UP_KW_MOLECULAR_FUNCTION | Myosin | 12 | 1.2 | 5.2E-5 |
| GOTERM_BP_DIRECT | integrin-mediated signaling pathway | 17 | 1.7 | 5.3E-5 |
| GOTERM_MF_DIRECT | calmodulin binding | 25 | 2.5 | 5.3E-5 |
| UP_KW_CELLULAR_COMPONENT | Secreted | 141 | 14.0 | 5.4E-5 |
| GOTERM_CC_DIRECT | myosin complex | 11 | 1.1 | 5.8E-5 |
| UP_KW_MOLECULAR_FUNCTION | Kinase | 65 | 6.4 | 5.9E-5 |
| GOTERM_BP_DIRECT | in utero embryonic development | 25 | 2.5 | 6.0E-5 |
| SMART | Int_alpha (Integrin alpha (beta-propellor repeats)) | 8 | 0.8 | 6.1E-5 |
| GOTERM_BP_DIRECT | sarcomere organization | 10 | 1.0 | 7.1E-5 |
| SMART | MYSc (Myosin. Large ATPases) | 11 | 1.1 | 7.4E-5 |
| GOTERM_CC_DIRECT | stress fiber | 13 | 1.3 | 8.9E-5 |
| UP_KW_MOLECULAR_FUNCTION | Heparin-binding | 16 | 1.6 | 9.0E-5 |
| KEGG_PATHWAY | AGE-RAGE signaling pathway in diabetic complications | 17 | 1.7 | 9.4E-5 |
| KEGG_PATHWAY | Pathways in cancer | 51 | 5.1 | 9.5E-5 |
| KEGG_PATHWAY | Hypertrophic cardiomyopathy | 16 | 1.6 | 9.6E-5 |
| GOTERM_BP_DIRECT | actomyosin structure organization | 9 | 0.9 | 1.0E-4 |
| GOTERM_MF_DIRECT | fibronectin binding | 9 | 0.9 | 1.0E-4 |
| SMART | EGF (Epidermal growth factor-like domain) | 26 | 2.6 | 1.1E-4 |
| INTERPRO | Calponin homology domain | 14 | 1.4 | 1.2E-4 |
| INTERPRO | Insulin-like growth factor binding protein, N-terminal | 19 | 1.9 | 1.2E-4 |
| GOTERM_BP_DIRECT | collagen-activated tyrosine kinase receptor signaling pathway | 6 | 0.6 | 1.3E-4 |
| INTERPRO | Integrin alpha chain | 7 | 0.7 | 1.4E-4 |
| INTERPRO | Integrin alpha-2 | 7 | 0.7 | 1.4E-4 |
| GOTERM_BP_DIRECT | cell-matrix adhesion | 16 | 1.6 | 1.5E-4 |
| GOTERM_CC_DIRECT | extracellular exosome | 137 | 13.6 | 1.5E-4 |
| GOTERM_BP_DIRECT | response to hypoxia | 20 | 2.0 | 1.6E-4 |
| UP_KW_CELLULAR_COMPONENT | Basement membrane | 10 | 1.0 | 1.6E-4 |
| GOTERM_BP_DIRECT | positive regulation of cholesterol efflux | 8 | 0.8 | 1.8E-4 |
| GOTERM_BP_DIRECT | protein phosphorylation | 42 | 4.2 | 1.9E-4 |
| GOTERM_BP_DIRECT | positive regulation of cell-substrate adhesion | 9 | 0.9 | 2.0E-4 |
| GOTERM_CC_DIRECT | platelet alpha granule lumen | 12 | 1.2 | 2.0E-4 |
| GOTERM_BP_DIRECT | muscle contraction | 14 | 1.4 | 2.0E-4 |
| UP_KW_DISEASE | Ehlers-Danlos syndrome | 7 | 0.7 | 2.7E-4 |
| INTERPRO | Complement Clr-like EGF domain | 8 | 0.8 | 2.8E-4 |
| INTERPRO | Pleckstrin homology domain | 28 | 2.8 | 3.7E-4 |
| KEGG_PATHWAY | Small cell lung cancer | 15 | 1.5 | 4.4E-4 |
| KEGG_PATHWAY | PI3K-Akt signaling pathway | 36 | 3.6 | 4.5E-4 |
| GOTERM_BP_DIRECT | cell adhesion mediated by integrin | 9 | 0.9 | 5.1E-4 |
| INTERPRO | Laminin G domain | 11 | 1.1 | 5.3E-4 |
| KEGG_PATHWAY | p53 signaling pathway | 13 | 1.3 | 5.4E-4 |
| INTERPRO | Protein kinase-like domain | 45 | 4.5 | 5.7E-4 |
| GOTERM_BP_DIRECT | muscle filament sliding | 6 | 0.6 | 6.0E-4 |
| INTERPRO | Protein kinase, catalytic domain | 42 | 4.2 | 6.4E-4 |
| INTERPRO | Integrin alpha chain, C-terminal cytoplasmic region, conserved site | 6 | 0.6 | 7.1E-4 |
| GOTERM_MF_DIRECT | protein serine/threonine/tyrosine kinase activity | 38 | 3.8 | 7.3E-4 |
| INTERPRO | von Willebrand factor, type C | 9 | 0.9 | 7.5E-4 |
| INTERPRO | Diacylglycerol/phorbol-ester binding | 7 | 0.7 | 7.7E-4 |
| INTERPRO | Peptidase M12B, ADAM-TS | 7 | 0.7 | 7.7E-4 |
| GOTERM_BP_DIRECT | hemopoiesis | 11 | 1.1 | 7.7E-4 |
| GOTERM_BP_DIRECT | chondrocyte differentiation | 10 | 1.0 | 7.8E-4 |
| KEGG_PATHWAY | Proteoglycans in cancer | 24 | 2.4 | 7.9E-4 |
| GOTERM_CC_DIRECT | Z disc | 16 | 1.6 | 9.0E-4 |
| UP_KW_CELLULAR_COMPONENT | Thick filament | 6 | 0.6 | 9.5E-4 |
| GOTERM_BP_DIRECT | cellular response to transforming growth factor beta stimulus | 11 | 1.1 | 9.9E-4 |
| UP_KW_DOMAIN | Coiled coil | 174 | 17.2 | 1.0E-3 |
| GOTERM_BP_DIRECT | regulation of cell growth | 12 | 1.2 | 1.1E-3 |
| KEGG_PATHWAY | Platelet activation | 17 | 1.7 | 1.1E-3 |
| GOTERM_BP_DIRECT | kidney development | 15 | 1.5 | 1.1E-3 |
| GOTERM_BP_DIRECT | Notch signaling pathway | 15 | 1.5 | 1.1E-3 |
| SMART | FN3 (Fibronectin type 3 domain) | 20 | 2.0 | 1.2E-3 |
| GOTERM_CC_DIRECT | cell-cell junction | 20 | 2.0 | 1.2E-3 |
| UP_KW_BIOLOGICAL_PROCESS | Angiogenesis | 17 | 1.7 | 1.2E-3 |
| INTERPRO | ADAM-TS Spacer 1 | 7 | 0.7 | 1.2E-3 |
| GOTERM_BP_DIRECT | angiogenesis | 24 | 2.4 | 1.2E-3 |
| SMART | VWC (von Willebrand factor (vWF) type C domain) | 9 | 0.9 | 1.3E-3 |
| GOTERM_MF_DIRECT | platelet-derived growth factor binding | 5 | 0.5 | 1.3E-3 |
| UP_KW_LIGAND | ATP-binding | 110 | 10.9 | 1.3E-3 |
| INTERPRO | Fibronectin, type III | 22 | 2.2 | 1.4E-3 |
| GOTERM_CC_DIRECT | integrin complex | 7 | 0.7 | 1.4E-3 |
| INTERPRO | Serine/threonine-protein kinase, active site | 29 | 2.9 | 1.4E-3 |
| SMART | CH (Calponin homology domain) | 12 | 1.2 | 1.4E-3 |
| GOTERM_BP_DIRECT | superoxide metabolic process | 6 | 0.6 | 1.4E-3 |
| GOTERM_BP_DIRECT | regulation of sodium ion transport | 6 | 0.6 | 1.4E-3 |
| GOTERM_CC_DIRECT | actomyosin | 5 | 0.5 | 1.6E-3 |
| GOTERM_BP_DIRECT | response to xenobiotic stimulus | 24 | 2.4 | 1.6E-3 |
| KEGG_PATHWAY | Regulation of actin cytoskeleton | 25 | 2.5 | 1.6E-3 |
| GOTERM_BP_DIRECT | elastic fiber assembly | 5 | 0.5 | 1.7E-3 |
| GOTERM_MF_DIRECT | metal ion binding | 161 | 16.0 | 1.8E-3 |
| GOTERM_BP_DIRECT | regulation of phosphatidylinositol 3-kinase activity | 4 | 0.4 | 1.8E-3 |
| GOTERM_BP_DIRECT | focal adhesion assembly | 7 | 0.7 | 1.9E-3 |
| GOTERM_BP_DIRECT | heart development | 21 | 2.1 | 1.9E-3 |
| GOTERM_CC_DIRECT | cytoskeleton | 39 | 3.9 | 1.9E-3 |
| GOTERM_CC_DIRECT | extracellular space | 116 | 11.5 | 1.9E-3 |
| UP_KW_CELLULAR_COMPONENT | Cell junction | 34 | 3.4 | 2.0E-3 |
| GOTERM_BP_DIRECT | positive regulation of ERK1 and ERK2 cascade | 22 | 2.2 | 2.0E-3 |
| GOTERM_BP_DIRECT | osteoblast differentiation | 15 | 1.5 | 2.1E-3 |
| GOTERM_MF_DIRECT | motor activity | 10 | 1.0 | 2.2E-3 |
| GOTERM_CC_DIRECT | cell surface | 46 | 4.6 | 2.3E-3 |
| INTERPRO | Protein kinase, ATP binding site | 33 | 3.3 | 2.3E-3 |
| GOTERM_BP_DIRECT | activation of GTPase activity | 15 | 1.5 | 2.3E-3 |
| GOTERM_BP_DIRECT | positive regulation of pri-miRNA transcription from RNA polymerase II promoter | 9 | 0.9 | 2.6E-3 |
| GOTERM_BP_DIRECT | regulation of cell proliferation | 18 | 1.8 | 2.6E-3 |
| KEGG_PATHWAY | Vascular smooth muscle contraction | 17 | 1.7 | 2.6E-3 |
| GOTERM_MF_DIRECT | protein kinase activity | 32 | 3.2 | 2.8E-3 |
| GOTERM_BP_DIRECT | skin development | 8 | 0.8 | 2.9E-3 |
| INTERPRO | IQ motif, EF-hand binding site | 12 | 1.2 | 3.2E-3 |
| GOTERM_BP_DIRECT | positive regulation of bone mineralization | 8 | 0.8 | 3.4E-3 |
| GOTERM_BP_DIRECT | inflammatory response | 33 | 3.3 | 3.4E-3 |
| BIOCARTA | Complement Pathway | 7 | 0.7 | 3.4E-3 |
| GOTERM_CC_DIRECT | adherens junction | 18 | 1.8 | 3.5E-3 |
| KEGG_PATHWAY | Amoebiasis | 14 | 1.4 | 3.6E-3 |
| GOTERM_MF_DIRECT | extracellular matrix binding | 7 | 0.7 | 3.8E-3 |
| GOTERM_BP_DIRECT | skeletal system development | 15 | 1.5 | 3.8E-3 |
| KEGG_PATHWAY | Phagosome | 18 | 1.8 | 3.8E-3 |
| GOTERM_CC_DIRECT | muscle myosin complex | 5 | 0.5 | 3.9E-3 |
| GOTERM_CC_DIRECT | nuclear membrane | 22 | 2.2 | 3.9E-3 |
| INTERPRO | Actinin-type, actin-binding, conserved site | 6 | 0.6 | 4.1E-3 |
| GOTERM_MF_DIRECT | structural constituent of muscle | 8 | 0.8 | 4.1E-3 |
| GOTERM_BP_DIRECT | positive regulation of protein metabolic process | 6 | 0.6 | 4.3E-3 |
| GOTERM_CC_DIRECT | membrane | 202 | 20.0 | 4.4E-3 |
| BIOCARTA | Classical Complement Pathway | 6 | 0.6 | 4.6E-3 |
| INTERPRO | Peptidase A1 | 5 | 0.5 | 4.7E-3 |
| UP_KW_PTM | Zymogen | 23 | 2.3 | 4.9E-3 |
| UP_KW_MOLECULAR_FUNCTION | Mitogen | 8 | 0.8 | 5.0E-3 |
| INTERPRO | Spectrin repeat | 6 | 0.6 | 5.0E-3 |
| GOTERM_BP_DIRECT | intrinsic apoptotic signaling pathway in response to endoplasmic reticulum stress | 7 | 0.7 | 5.0E-3 |
| INTERPRO | PKD/Chitinase domain | 4 | 0.4 | 5.2E-3 |
| GOTERM_BP_DIRECT | positive regulation of protein autophosphorylation | 6 | 0.6 | 5.2E-3 |
| GOTERM_BP_DIRECT | ventricular cardiac muscle tissue morphogenesis | 6 | 0.6 | 5.2E-3 |
| UP_KW_BIOLOGICAL_PROCESS | Complement pathway | 7 | 0.7 | 5.2E-3 |
| UP_KW_MOLECULAR_FUNCTION | Serine/threonine-protein kinase | 34 | 3.4 | 5.2E-3 |
|  |  |  |  |  |
| UP_KW_MOLECULAR_FUNCTION | Motor protein | 16 | 1.6 | 5.4E-3 |
| GOTERM_BP_DIRECT | positive regulation of cell migration | 23 | 2.3 | 5.5E-3 |
| GOTERM_BP_DIRECT | cellular response to amino acid stimulus | 9 | 0.9 | 5.7E-3 |
| GOTERM_MF_DIRECT | protein serine/threonine kinase activity | 32 | 3.2 | 5.7E-3 |
| GOTERM_BP_DIRECT | aortic valve morphogenesis | 7 | 0.7 | 5.8E-3 |
| INTERPRO | Immunoglobulin I-set | 15 | 1.5 | 5.8E-3 |
| GOTERM_BP_DIRECT | positive regulation of GTPase activity | 18 | 1.8 | 6.0E-3 |
| INTERPRO | DNA repair protein XRCC4, C-terminal | 5 | 0.5 | 6.0E-3 |
| GOTERM_CC_DIRECT | cortical actin cytoskeleton | 9 | 0.9 | 6.1E-3 |
| GOTERM_BP_DIRECT | positive regulation of focal adhesion assembly | 6 | 0.6 | 6.2E-3 |
| GOTERM_BP_DIRECT | regulation of cell adhesion | 9 | 0.9 | 6.3E-3 |
| INTERPRO | SH2 domain | 13 | 1.3 | 6.5E-3 |
| GOTERM_BP_DIRECT | intracellular signal transduction | 34 | 3.4 | 6.6E-3 |
| GOTERM_BP_DIRECT | actin filament-based movement | 5 | 0.5 | 6.7E-3 |
| GOTERM_BP_DIRECT | phospholipid homeostasis | 5 | 0.5 | 6.7E-3 |
| GOTERM_CC_DIRECT | specific granule lumen | 9 | 0.9 | 6.8E-3 |
| GOTERM_MF_DIRECT | ADP binding | 7 | 0.7 | 6.9E-3 |
| GOTERM_CC_DIRECT | actin filament | 11 | 1.1 | 7.1E-3 |
| GOTERM_MF_DIRECT | macromolecular complex binding | 29 | 2.9 | 7.1E-3 |
| GOTERM_BP_DIRECT | positive regulation of cell division | 8 | 0.8 | 7.1E-3 |
| GOTERM_BP_DIRECT | epithelial to mesenchymal transition | 8 | 0.8 | 7.1E-3 |
| SMART | PH (Pleckstrin homology domain) | 27 | 2.7 | 7.1E-3 |
| UP_KW_PTM | Hydroxylation | 21 | 2.1 | 7.1E-3 |
| INTERPRO | Platelet-derived growth factor (PDGF) | 4 | 0.4 | 7.5E-3 |
| INTERPRO | TB domain | 4 | 0.4 | 7.5E-3 |
| PIR_SUPERFAMILY | amyloid protein, SAA type | 3 | 0.3 | 7.7E-3 |
| KEGG_PATHWAY | Chronic myeloid leukemia | 11 | 1.1 | 8.1E-3 |
| GOTERM_BP_DIRECT | positive regulation of DNA biosynthetic process | 5 | 0.5 | 8.4E-3 |
| GOTERM_BP_DIRECT | positive regulation of osteoblast differentiation | 10 | 1.0 | 8.4E-3 |
| GOTERM_BP_DIRECT | positive regulation of collagen biosynthetic process | 6 | 0.6 | 8.6E-3 |
| SMART | PKD (Repeats in polycystic kidney disease 1 (PKD1) and other proteins) | 4 | 0.4 | 8.9E-3 |
| GOTERM_CC_DIRECT | cell projection | 17 | 1.7 | 8.9E-3 |
| UP_KW_DOMAIN | Signal | 255 | 25.3 | 9.1E-3 |
| GOTERM_BP_DIRECT | protein autophosphorylation | 17 | 1.7 | 9.3E-3 |
| SMART | TSPN (Thrombospondin N-terminal -like domains) | 6 | 0.6 | 9.3E-3 |
| GOTERM_BP_DIRECT | positive regulation of angiogenesis | 16 | 1.6 | 9.5E-3 |
| UP_KW_MOLECULAR_FUNCTION | Integrin | 8 | 0.8 | 9.5E-3 |
| GOTERM_BP_DIRECT | positive regulation of peptidyl-tyrosine phosphorylation | 11 | 1.1 | 9.5E-3 |
| GOTERM_BP_DIRECT | cell migration | 23 | 2.3 | 9.5E-3 |
| UP_KW_DOMAIN | SH2 domain | 13 | 1.3 | 9.6E-3 |
| UP_KW_PTM | Glycoprotein | 292 | 28.9 | 9.7E-3 |
| GOTERM_BP_DIRECT | positive regulation of epithelial to mesenchymal transition | 8 | 0.8 | 9.8E-3 |
| GOTERM_BP_DIRECT | receptor-mediated endocytosis | 10 | 1.0 | 9.9E-3 |
| GOTERM_BP_DIRECT | negative regulation of tumor necrosis factor production | 10 | 1.0 | 9.9E-3 |
| GOTERM_MF_DIRECT | L-amino acid transmembrane transporter activity | 6 | 0.6 | 1.0E-2 |
| GOTERM_MF_DIRECT | extracellular matrix constituent conferring elasticity | 4 | 0.4 | 1.0E-2 |

**Table S9.** RNA-Seq analysis. Functional annotation chart of downregulated genes (Post- *vs* Pre-flight; fold change cut-off, 2.0) in the astronaut’s muscle tissue. EASE Score, a modified Fisher Exact *P*-Value, for gene-enrichment analysis. *P* ≤0.01.

| Category | Term | Count | % | P-Value |
| --- | --- | --- | --- | --- |
| GOTERM_BP_DIRECT | cell-cell adhesion via plasma-membrane adhesion molecules | 5 | 3.8 | 6.1E-5 |
| GOTERM_MF_DIRECT | calcium ion binding | 15 | 11.5 | 1.4E-4 |
| SMART | EGF (Epidermal growth factor-like domain) | 7 | 5.4 | 1.3E-3 |
| GOTERM_CC_DIRECT | extracellular space | 23 | 17.7 | 1.7E-3 |
| GOTERM_BP_DIRECT | positive regulation of bone mineralization | 4 | 3.1 | 1.8E-3 |
| GOTERM_BP_DIRECT | calcium-dependent cell-cell adhesion via plasma membrane cell adhesion molecules | 4 | 3.1 | 2.5E-3 |
| GOTERM_BP_DIRECT | positive regulation of ossification | 3 | 2.3 | 2.7E-3 |
| INTERPRO | Epidermal growth factor-like domain | 7 | 5.4 | 2.8E-3 |
| GOTERM_BP_DIRECT | oxygen transport | 3 | 2.3 | 3.6E-3 |
| UP_KW_MOLECULAR_FUNCTION | Growth factor | 5 | 3.8 | 4.7E-3 |
| UP_KW_BIOLOGICAL_PROCESS | Cell adhesion | 9 | 6.9 | 5.7E-3 |
| UP_KW_DOMAIN | EGF-like domain | 7 | 5.4 | 6.4E-3 |
| INTERPRO | Cadherin, cytoplasmic domain | 3 | 2.3 | 7.0E-3 |
| GOTERM_BP_DIRECT | positive regulation of gene expression | 9 | 6.9 | 8.5E-3 |
| GOTERM_BP_DIRECT | positive regulation of osteoblast differentiation | 4 | 3.1 | 9.1E-3 |

**Table S10.** RNA-Seq analysis. Genes of the most significant upregulated terms (Post- *vs* Pre-flight; fold change cut-off, 2.0) in the astronaut’s muscle tissue.

| Term: Extracellular matrix | |
| --- | --- |
| GENE ID | **GENE NAME** |
| 9510 | ADAM metallopeptidase with thrombospondin type 1 motif 1(ADAMTS1) |
| 81792 | ADAM metallopeptidase with thrombospondin type 1 motif 12(ADAMTS12) |
| 9507 | ADAM metallopeptidase with thrombospondin type 1 motif 4(ADAMTS4) |
| 11096 | ADAM metallopeptidase with thrombospondin type 1 motif 5(ADAMTS5) |
| 56999 | ADAM metallopeptidase with thrombospondin type 1 motif 9(ADAMTS9) |
| 57188 | ADAMTS like 3(ADAMTSL3) |
| 54507 | ADAMTS like 4(ADAMTSL4) |
| 2202 | EGF containing fibulin extracellular matrix protein 1(EFEMP1) |
| 129080 | EMI domain containing 1(EMID1) |
| 6695 | SPARC (osteonectin), cwcv and kazal like domains proteoglycan 1(SPOCK1) |
| 64093 | SPARC related modular calcium binding 1(SMOC1) |
| 54361 | Wnt family member 4(WNT4) |
| 7483 | Wnt family member 9A(WNT9A) |
| 54829 | asporin(ASPN) |
| 8483 | cartilage intermediate layer protein(CILP) |
| 1490 | cellular communication network factor 2(CCN2) |
| 151887 | coiled-coil domain containing 80(CCDC80) |
| 115908 | collagen triple helix repeat containing 1(CTHRC1) |
| 1277 | collagen type I alpha 1 chain(COL1A1) |
| 1281 | collagen type III alpha 1 chain(COL3A1) |
| 1282 | collagen type IV alpha 1 chain(COL4A1) |
| 1284 | collagen type IV alpha 2 chain(COL4A2) |
| 1287 | collagen type IV alpha 5 chain(COL4A5) |
| 1290 | collagen type V alpha 2 chain(COL5A2) |
| 1291 | collagen type VI alpha 1 chain(COL6A1) |
| 1292 | collagen type VI alpha 2 chain(COL6A2) |
| 1310 | collagen type XIX alpha 1 chain(COL19A1) |
| 1307 | collagen type XVI alpha 1 chain(COL16A1) |
| 80781 | collagen type XVIII alpha 1 chain(COL18A1) |
| 2006 | elastin(ELN) |
| 2200 | fibrillin 1(FBN1) |
| 2199 | fibulin 2(FBLN2) |
| 10516 | fibulin 5(FBLN5) |
| 3339 | heparan sulfate proteoglycan 2(HSPG2) |
| 50939 | interphotoreceptor matrix proteoglycan 2(IMPG2) |
| 3898 | ladinin 1(LAD1) |
| 3910 | laminin subunit alpha 4(LAMA4) |
| 4053 | latent transforming growth factor beta binding protein 2(LTBP2) |
| 4054 | latent transforming growth factor beta binding protein 3(LTBP3) |
| 4017 | lysyl oxidase like 2(LOXL2) |
| 4811 | nidogen 1(NID1) |
| 10631 | periostin(POSTN) |
| 10417 | spondin 2(SPON2) |
| 3371 | tenascin C(TNC) |
| 7148 | tenascin XB(TNXB) |
| 7057 | thrombospondin 1(THBS1) |
| 7060 | thrombospondin 4(THBS4) |
| 7040 | transforming growth factor beta 1(TGFB1) |
| 7042 | transforming growth factor beta 2(TGFB2) |
| 7045 | transforming growth factor beta induced(TGFBI) |
| 7052 | transglutaminase 2(TGM2) |
|  | |
| Term: Cell adhesion | |
| GENE ID | **GENE NAME** |
| 27 | ABL proto-oncogene 2, non-receptor tyrosine kinase(ABL2) |
| 421 | ARVCF delta catenin family member(ARVCF) |
| 6347 | C-C motif chemokine ligand 2(CCL2) |
| 6376 | C-X3-C motif chemokine ligand 1(CX3CL1) |
| 10085 | EGF like repeats and discoidin domains 3(EDIL3) |
| 50848 | F11 receptor(F11R) |
| 2195 | FAT atypical cadherin 1(FAT1) |
| 341640 | FRAS1 related extracellular matrix 2(FREM2) |
| 57326 | PBX homeobox interacting protein 1(PBXIP1) |
| 6695 | SPARC (osteonectin), cwcv and kazal like domains proteoglycan 1(SPOCK1) |
| 7130 | TNF alpha induced protein 6(TNFAIP6) |
| 51330 | TNF receptor superfamily member 12A(TNFRSF12A) |
| 7070 | Thy-1 cell surface antigen(THY1) |
| 88 | actinin alpha 2(ACTN2) |
| 26574 | apoptosis antagonizing transcription factor(AATF) |
| 1013 | cadherin 15(CDH15) |
| 1002 | cadherin 4(CDH4) |
| 22883 | calsyntenin 1(CLSTN1) |
| 50937 | cell adhesion associated, oncogene regulated(CDON) |
| 3491 | cellular communication network factor 1(CCN1) |
| 1490 | cellular communication network factor 2(CCN2) |
| 100132463 | claudin 24(CLDN24) |
| 1277 | collagen type I alpha 1 chain(COL1A1) |
| 1291 | collagen type VI alpha 1 chain(COL6A1) |
| 1292 | collagen type VI alpha 2 chain(COL6A2) |
| 1310 | collagen type XIX alpha 1 chain(COL19A1) |
| 1307 | collagen type XVI alpha 1 chain(COL16A1) |
| 80781 | collagen type XVIII alpha 1 chain(COL18A1) |
| 8506 | contactin associated protein 1(CNTNAP1) |
| 1803 | dipeptidyl peptidase 4(DPP4) |
| 780 | discoidin domain receptor tyrosine kinase 1(DDR1) |
| 4921 | discoidin domain receptor tyrosine kinase 2(DDR2) |
| 10457 | glycoprotein nmb(GPNMB) |
| 22997 | immunoglobulin superfamily member 9B(IGSF9B) |
| 8515 | integrin subunit alpha 10(ITGA10) |
| 3673 | integrin subunit alpha 2(ITGA2) |
| 3675 | integrin subunit alpha 3(ITGA3) |
| 3678 | integrin subunit alpha 5(ITGA5) |
| 8516 | integrin subunit alpha 8(ITGA8) |
| 3680 | integrin subunit alpha 9(ITGA9) |
| 3684 | integrin subunit alpha M(ITGAM) |
| 3384 | intercellular adhesion molecule 2(ICAM2) |
| 3910 | laminin subunit alpha 4(LAMA4) |
| 10894 | lymphatic vessel endothelial hyaluronan receptor 1(LYVE1) |
| 4017 | lysyl oxidase like 2(LOXL2) |
| 4608 | myosin binding protein H(MYBPH) |
| 4684 | neural cell adhesion molecule 1(NCAM1) |
| 4739 | neural precursor cell expressed, developmentally downregulated 9(NEDD9) |
| 23114 | neurofascin(NFASC) |
| 57555 | neuroligin 2(NLGN2) |
| 84033 | obscurin, cytoskeletal calmodulin and titin-interacting RhoGEF(OBSCN) |
| 10631 | periostin(POSTN) |
| 25865 | protein kinase D2(PRKD2) |
| 5796 | protein tyrosine phosphatase receptor type K(PTPRK) |
| 5802 | protein tyrosine phosphatase receptor type S(PTPRS) |
| 56123 | protocadherin beta 13(PCDHB13) |
| 56099 | protocadherin gamma subfamily B, 7(PCDHGB7) |
| 6404 | selectin P ligand(SELPLG) |
| 10417 | spondin 2(SPON2) |
| 79987 | sushi, von Willebrand factor type A, EGF and pentraxin domain containing 1(SVEP1) |
| 8189 | symplekin scaffold protein(SYMPK) |
| 7094 | talin 1(TLN1) |
| 3371 | tenascin C(TNC) |
| 7148 | tenascin XB(TNXB) |
| 7057 | thrombospondin 1(THBS1) |
| 7058 | thrombospondin 2(THBS2) |
| 7060 | thrombospondin 4(THBS4) |
| 7045 | transforming growth factor beta induced(TGFBI) |
|  | |
| Term: Integrin binding | |
| GENE ID | **GENE NAME** |
| 11096 | ADAM metallopeptidase with thrombospondin type 1 motif 5(ADAMTS5) |
| 6376 | C-X3-C motif chemokine ligand 1(CX3CL1) |
| 10085 | EGF like repeats and discoidin domains 3(EDIL3) |
| 50848 | F11 receptor(F11R) |
| 2674 | GDNF family receptor alpha 1(GFRA1) |
| 7070 | Thy-1 cell surface antigen(THY1) |
| 87 | actinin alpha 1(ACTN1) |
| 88 | actinin alpha 2(ACTN2) |
| 3491 | cellular communication network factor 1(CCN1) |
| 1490 | cellular communication network factor 2(CCN2) |
| 1281 | collagen type III alpha 1 chain(COL3A1) |
| 1307 | collagen type XVI alpha 1 chain(COL16A1) |
| 2200 | fibrillin 1(FBN1) |
| 10516 | fibulin 5(FBLN5) |
| 10457 | glycoprotein nmb(GPNMB) |
| 3339 | heparan sulfate proteoglycan 2(HSPG2) |
| 3479 | insulin like growth factor 1(IGF1) |
| 8515 | integrin subunit alpha 10(ITGA10) |
| 3673 | integrin subunit alpha 2(ITGA2) |
| 3675 | integrin subunit alpha 3(ITGA3) |
| 3678 | integrin subunit alpha 5(ITGA5) |
| 8516 | integrin subunit alpha 8(ITGA8) |
| 3680 | integrin subunit alpha 9(ITGA9) |
| 3684 | integrin subunit alpha M(ITGAM) |
| 3384 | intercellular adhesion molecule 2(ICAM2) |
| 4627 | myosin heavy chain 9(MYH9) |
| 11188 | nischarin(NISCH) |
| 6423 | secreted frizzled related protein 2(SFRP2) |
| 1903 | sphingosine-1-phosphate receptor 3(S1PR3) |
| 7094 | talin 1(TLN1) |
| 7148 | tenascin XB(TNXB) |
| 7057 | thrombospondin 1(THBS1) |
| 7060 | thrombospondin 4(THBS4) |
| 7045 | transforming growth factor beta induced(TGFBI) |
|  | |
| Term: Myosin filament | |
| GENE ID | **GENE NAME** |
| 72 | actin gamma 2, smooth muscle(ACTG2) |
| 399687 | myosin XVIIIA(MYO18A) |
| 4608 | myosin binding protein H(MYBPH) |
| 4629 | myosin heavy chain 11(MYH11) |
| 79784 | myosin heavy chain 14(MYH14) |
| 4624 | myosin heavy chain 6(MYH6) |
| 4625 | myosin heavy chain 7(MYH7) |
| 57644 | myosin heavy chain 7B(MYH7B) |
| 4626 | myosin heavy chain 8(MYH8) |
| 4627 | myosin heavy chain 9(MYH9) |
|  | |
| Term: Muscle protein | |
| GENE ID | **GENE NAME** |
| 70 | actin alpha cardiac muscle 1(ACTC1) |
| 72 | actin gamma 2, smooth muscle(ACTG2) |
| 845 | calsequestrin 2(CASQ2) |
| 4608 | myosin binding protein H(MYBPH) |
| 4629 | myosin heavy chain 11(MYH11) |
| 4624 | myosin heavy chain 6(MYH6) |
| 4625 | myosin heavy chain 7(MYH7) |
| 57644 | myosin heavy chain 7B(MYH7B) |
| 4626 | myosin heavy chain 8(MYH8) |
| 140465 | myosin light chain 6B(MYL6B) |
| 84033 | obscurin, cytoskeletal calmodulin and titin-interacting RhoGEF(OBSCN) |
| 6876 | transgelin(TAGLN) |
| 7171 | tropomyosin 4(TPM4) |
| 7138 | troponin T1, slow skeletal type(TNNT1) |
| 7139 | troponin T2, cardiac type(TNNT2) |

**Table S11.** RNA-Seq analysis. Genes of the most significant downregulated terms (Post- *vs* Pre-flight; fold change cut-off, 2.0) in the astronaut’s muscle tissue.

| Term: Cell-cell adhesion via plasma-membrane adhesion molecules | |
| --- | --- |
| GENE ID | **GENE NAME** |
| 57126 | CD177 molecule(CD177) |
| 28513 | cadherin 19(CDH19) |
| 28316 | cadherin 20(CDH20) |
| 1005 | cadherin 7(CDH7) |
| 4359 | myelin protein zero(MPZ) |
|  | |
| Term: Calcium ion binding | |
| GENE ID | **GENE NAME** |
| 6285 | S100 calcium binding protein B(S100B) |
| 28513 | cadherin 19(CDH19) |
| 28316 | cadherin 20(CDH20) |
| 1005 | cadherin 7(CDH7) |
| 10518 | calcium and integrin binding family member 2(CIB2) |
| 91860 | calmodulin like 4(CALML4) |
| 8788 | delta like non-canonical Notch ligand 1(DLK1) |
| 4147 | matrilin 2(MATN2) |
| 4151 | myoglobin(MB) |
| 9378 | neurexin 1(NRXN1) |
| 5816 | parvalbumin(PVALB) |
| 5100 | protocadherin 8(PCDH8) |
| 57758 | signal peptide, CUB domain and EGF like domain containing 2(SCUBE2) |
| 57451 | teneurin transmembrane protein 2(TENM2) |
| 7178 | tumor protein, translationally-controlled 1(TPT1) |
|  | |
| Term: EGF | |
| GENE ID | **GENE NAME** |
| 4041 | LDL receptor related protein 5(LRP5) |
| 8788 | delta like non-canonical Notch ligand 1(DLK1) |
| 4147 | matrilin 2(MATN2) |
| 145957 | neuregulin 4(NRG4) |
| 9378 | neurexin 1(NRXN1) |
| 57758 | signal peptide, CUB domain and EGF like domain containing 2(SCUBE2) |
| 57451 | teneurin transmembrane protein 2(TENM2) |
|  | |
| Term: Extracellular space | |
| GENE ID | **GENE NAME** |
| 4283 | C-X-C motif chemokine ligand 9(CXCL9) |
| 6285 | S100 calcium binding protein B(S100B) |
| 563 | alpha-2-glycoprotein 1, zinc-binding(AZGP1) |
| 10218 | angiopoietin like 7(ANGPTL7) |
| 347 | apolipoprotein D(APOD) |
| 655 | bone morphogenetic protein 7(BMP7) |
| 1270 | ciliary neurotrophic factor(CNTF) |
| 1299 | collagen type IX alpha 3 chain(COL9A3) |
| 8788 | delta like non-canonical Notch ligand 1(DLK1) |
| 2246 | fibroblast growth factor 1(FGF1) |
| 83888 | fibroblast growth factor binding protein 2(FGFBP2) |
| 3043 | hemoglobin subunit beta(HBB) |
| 116135 | leucine rich repeat containing 3B(LRRC3B) |
| 4060 | lumican(LUM) |
| 692094 | microseminoprotein, prostate associated(MSMP) |
| 145957 | neuregulin 4(NRG4) |
| 5764 | pleiotrophin(PTN) |
| 51435 | scavenger receptor class A member 3(SCARA3) |
| 6425 | secreted frizzled related protein 5(SFRP5) |
| 57758 | signal peptide, CUB domain and EGF like domain containing 2(SCUBE2) |
| 11341 | stimulator of chondrogenesis 1(SCRG1) |
| 7178 | tumor protein, translationally-controlled 1(TPT1) |
| 284013 | vitelline membrane outer layer 1 homolog(VMO1) |
|  | |
| Term: Positive regulation of bone mineralization | |
| GENE ID | **GENE NAME** |
| 655 | bone morphogenetic protein 7(BMP7) |
| 658 | bone morphogenetic protein receptor type 1B(BMPR1B) |
| 5764 | pleiotrophin(PTN) |
| 7020 | transcription factor AP-2 alpha(TFAP2A) |
